# Detection of TP53 dysfunction in chronic lymphocytic leukemia by an in vitro functional assay based on TP53 activation by the non-genotoxic drug Nutlin-3: a proposal for clinical application

**DOI:** 10.1186/1756-8722-6-83

**Published:** 2013-11-05

**Authors:** Federico Pozzo, Michele Dal Bo, Nadia Peragine, Riccardo Bomben, Antonella Zucchetto, Francesca Maria Rossi, Massimo Degan, Davide Rossi, Annalisa Chiarenza, Alberto Grossi, Francesco Di Raimondo, Francesco Zaja, Gabriele Pozzato, Paola Secchiero, Gianluca Gaidano, Giovanni Del Poeta, Giorgio Zauli, Robin Foà, Anna Guarini, Valter Gattei

**Affiliations:** 1 Clinical and Experimental Onco-Hematology Unit, Centro di Riferimento Oncologico, I.R.C.C.S., Via Franco Gallini 2, Aviano, (PN), Italy; 2 Division of Hematology, Department of Cellular Biotechnologies and Hematology, Sapienza University, Rome, Italy; 3 Division of Hematology, Department of Translational Medicine, Amedeo Avogadro University of Eastern Piedmont, Novara, Italy; 4 Division of Hematology, Ferrarotto Hospital, Catania, Italy; 5 Hematology, IFCA, Firenze, Italy; 6 Clinica Ematologica, Centro Trapianti e Terapie Cellulari “Carlo Melzi” DISM, Azienda Ospedaliera Universitaria S. Maria Misericordia, Udine, Italy; 7 Department of Internal Medicine and Hematology, Maggiore General Hospital, University of Trieste, Trieste, Italy; 8 Department of Morphology and Embryology, Human Anatomy Section, University of Ferrara, Ferrara, Italy; 9 Division of Hematology, S.Eugenio Hospital and University of Tor Vergata, Rome, Italy; 10 Institute for Maternal and Child Health - IRCCS “Burlo Garofolo”, Trieste, Italy

**Keywords:** CLL, TP53, Prognosis

## Abstract

**Background:**

*TP53* defects, i.e. 17p13 deletion and/or nucleotide mutations, associate with short survival and chemorefractoriness in chronic lymphocytic leukemia (CLL). In this context, since direct sequencing of the *TP53* gene does not evaluate TP53 functionality, a functional assessment of TP53 pathway may be of interest to identify high risk CLL. By taking advantage of a training cohort of 100 CLL and a validation cohort of 40 CLL with different patterns of *TP53* mutation/deletion by FISH and sequencing, we propose an in-vitro assay in which the modulation of TP53 protein and *CDKN1A* mRNA were investigated upon 24-hour exposure of CLL cells to Nutlin-3.

**Methods:**

The functional assay was set-up on cell lines recapitulating all *TP53* genotypes (EHEB, *TP53*^wt/wt^; RAJI, *TP53*^mut/wt^; MEC-1 and MAVER1, *TP53*^mut/del^; HL-60, *TP53*^del/del^) and evaluated in two multi-institutional cohorts, purposely enriched in CLL bearing *TP53* disruption: a training cohort of 100 cases and a validation cohort of 40 cases, both characterized by FISH and *TP53* direct sequencing. Cells were exposed to 10 μM Nutlin-3 for 24 hours; TP53 accumulation was evaluated by Western blotting; TP53 transcriptional activity was determined by quantitative realtime PCR (qRT-PCR) of the TP53 target gene *CDKN1A*.

**Results:**

According to TP53 protein modulation, in the training cohort we identified: i) 63 cases (51 *TP53*^wt/wt^, 12 *TP53*^del/wt^) with absence of basal TP53 and induction after treatment (normal pattern); ii) 18 cases (3 *TP53*^mut/wt^, 15 *TP53*^mut/del^) with high basal TP53 without increase after treatment (mutant pattern); iii) 19 cases (5 *TP53*^mut/wt^; 3 *TP53*^mut/del^; 11 *TP53*^wt/wt^) with basal TP53 that increases upon treatment (intermediate pattern). Evaluation of *CDKN1A* mRNA levels upon Nutlin-3 exposure showed that the 26 *TP53* mutated (*TP53*^mut/del^ or *TP53*^mut/wt^) cases had lower induction levels than the majority (57/63) of cases with normal pattern, and 10/12 cases with intermediate pattern without evidence of TP53 derangement by FISH and sequencing. These results were confirmed in the independent validation cohort of 40 cases (13 *TP53*^wt/wt^, 3 *TP53*^del/wt^, 12 *TP53*^mut/del^, 12 *TP53*^mut/wt^).

**Conclusions:**

The proposed functional assay may integrate the conventional analyses for the identification of TP53 dysregulated CLL.

## Background

Chronic lymphocytic leukemia (CLL) is a heterogeneous disease with highly variable clinical courses with survivals ranging from months to decades [1]. In particular, a subset of patients is affected by a high-risk CLL form that rapidly progresses and develops a symptomatic disease requiring treatment. Over-represented in this group are patients bearing either a chromosomal deletion of 17p13.1, location of the tumor suppressor gene *TP53*, and/or carrying mutations of the *TP53* gene [2-4]. Over 80% of CLL with a deletion at 17p13 also present a *TP53* mutation in the remaining allele, whereas *TP53* mutations in absence of a concomitant deletion at 17p13 occur in 5-10% of CLL cases [5-9].

The TP53 protein is a transcription factor with a short half-life, present at low levels under resting conditions and that becomes activated following DNA damage. Activation, occurring predominantly by phosphoryilation, prolongs the half-life of the protein and allows it to accumulate into the nucleus where it induces apoptosis, cell cycle arrest, and DNA repair [10], thus playing a pivotal role in limiting clonal expansion, maintaining genomic stability, eventually mediating the action of DNA damaging chemotherapy [11-15]. Conventional treatment of CLL is usually based on cytotoxic chemotherapy using alkylating agents or nucleoside analogues. The group of patients bearing a TP53 disruption (i.e. deletion of 17p13 and mutations of the *TP53* gene, or *TP53* gene mutations alone) has been shown to respond particularly poorly to chemotherapy [2-4,16,17]. Therefore, although deletion or mutation of *TP53* gene in previously untreated CLL patients are reported to be 10-15% [7,8,18], the frequency of TP53 dysfunction increases to nearly 50% of patients when the disease progresses following initial therapies [19,20], suggesting that DNA damaging therapies exert a selective pressure that may lead to TP53 inactivation and subsequent resistance to commonly used chemotherapeutic agents.

In the last decade, functional assays in primary CLL cells have been developed [20-29], with the aim: i) to avoid large time- and money-consuming screenings of *TP53* gene mutations in non-17p deleted CLL cases; ii) to detect defects in the TP53 pathway escaping fluorescence-in situ-hybridization (FISH) for 17p deletions or mutational analysis by direct sequencing. In particular, in-vitro exposure of CLL cells to the small non-genotoxic molecule Nutlin-3, a potent and selective inhibitor of TP53/MDM2 interaction, has been proposed to evaluate TP53 functionality [21,26,30].

By applying a training/validation strategy using a cohort of 140 CLL cases with known TP53 status, we propose a short term in vitro functional assay, based on the exposure of CLL cells to the non-genotoxic TP53 activator Nutlin-3, as a tool to identify CLL cases with dysregulated TP53 in a clinical setting.

## Results

### Set up of a western blot assay to detect TP53 dysfunctions

A series of 5 cell lines encompassing all the types of TP53 dysfunction was employed: i) EHEB cells lacking both 17p deletion and *TP53* mutations (*TP53*^wt/wt^); ii) RAJI cells carrying *TP53* mutations in the absence of concomitant 17p deletion (*TP53*^mut/wt^); iii) MAVER-1 and MEC-1 cells carrying both 17p deletion and *TP53* mutations (*TP53*^del/mut^); iv) HL-60 cells bearing 17p deletion in both alleles (*TP53*^del/del^). The *TP53* status of the employed cell clones was re-confirmed in the present study by both FISH and direct sequencing approaches in agreement with data reported by the IARC *TP53* Mutation Database [31] (Additional file 1: Table S1).

**Figure 1:**
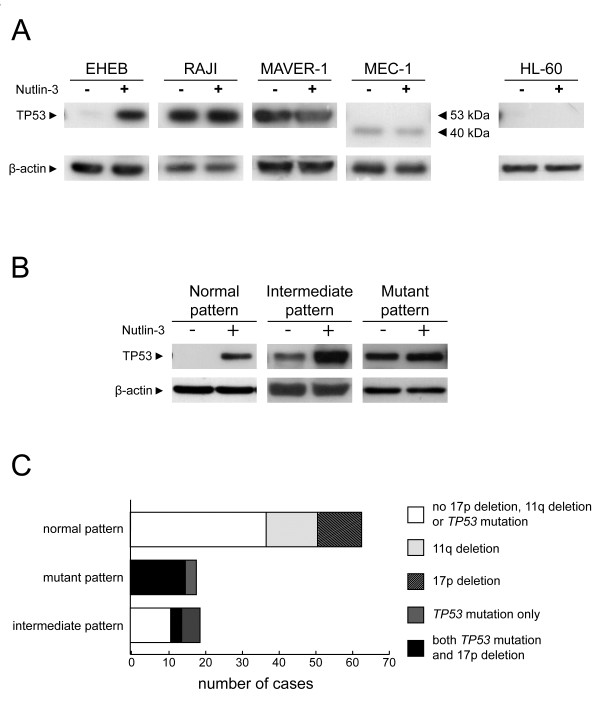
**Set up of a western blot assay to detect TP53 dysfunction. A)** Figure shows results from western blot assay on the series of the 5 cell lines, in particular EHEB (*TP53*^wt/wt^) with a normal pattern, MEC-1 (*TP53*^del/mut^), MAVER-1 (*TP53*^del/mut^), RAJI (*TP53*^mut/wt^) with a mutant pattern, and HL60 (*TP53*^del/del^) with a null pattern. **B)** Figure shows prototypic results from western blot assay of CLL cases with a normal pattern, with an intermediate pattern and with a mutant pattern. **C)** Histograms show the functional classification according to western blot assay of CLL cases of the training cohort subdivided into the main genetic subgroups. For each functional category the subdivision in genetic subgroup is indicated.

Cells from cell lines bearing or not TP53 dysfunction were treated for 24 hours with 10 μM Nutlin-3, and the levels of TP53 were evaluated by western blotting. As summarized in Figure 1A, EHEB cells (*TP53*^wt/wt^) showed an absence of basal TP53 and a marked induction upon in vitro Nutlin-3 exposure (“normal” pattern). An AnnexinV/7-AAD assay verified that Nutlin-3 exposure was capable to effectively induce apoptosis in EHEB cells, as documented by the death of the majority of the population within 24 hours (Additional file 2: Figure S1). In contrast, MAVER-1 (*TP53*^del/mut^), RAJI (*TP53*^mut/wt^) and MEC-1 (*TP53*^del/mut^) cells showed a pattern characterized by comparable TP53 levels between Nutlin-3 treated and untreated cells (“mutant” pattern; Figure 1A), mapping at the conventional weight for MAVER-1 and RAJI, or at lower molecular weight for MEC-1 cells, which expressed a truncated TP53 protein, as reported [31]. Finally, a total absence of TP53 levels before and upon in vitro Nutlin-3 exposure was detected in HL-60 cells (*TP53*^del/del^; “null” pattern; Figure 1A). Consistently, comparable mortality rates between Nutlin-3 treated and untreated conditions were observed by AnnexinV/7-AAD assay in cell lines expressing mutant/null TP53 statuses by western blot (Additional file 2: Figure S1).

To evaluate the sensitivity of the western blot assay for TP53 detection, RAJI cells, expressing TP53 in basal conditions, were mixed with EHEB cells, not expressing TP53 in basal conditions, in order to obtain samples containing 1%, 5%, 10%, 20%, 50% and 100% of RAJI cells. A conservative estimate based on western blot results suggested a sensitivity capable to detect as low as 5% of TP53-expressing cells (Additional file 2: Figure S1B).

### Functional characterization of TP53 dysfunction in CLL cases

In order to test the capability of the western blot assay to detect TP53 functionality, we employed a training cohort of 100 CLL cases. This cohort was purposely enriched in cases bearing *TP53* disruption (38 out of 100; 38%), including 17 p deletion alone (*TP53*^del/wt^, 12 cases; 12%), 17p deletion associated with concomitant *TP53* mutations (*TP53*^del/mut^, 18 cases; 18%), and a *TP53* mutated status in the absence of 17p deletion (*TP53*^mut/wt^, 8 cases; 8%), as indicated by FISH and direct sequencing approaches (Additional file 1: Table S2). In particular, FISH analyses revealed a percentage of 17p deleted nuclei ranging from 14% to 90% in the *TP53* mutated cases (median percentage 53% of deleted nuclei) and from 5% to 70% in deleted only cases (median percentage 10% of deleted nuclei, p = 0.003). Considering the evaluation of *TP53* mutational status by direct sequencing, all *TP53* mutations clustered in exons 5–8 with the only exclusion of a case, in which a mutation in intron 3 was detected (Additional file 1: Table S2).

By performing western blot analysis of TP53 expression on CLL cells from the training cohort exposed or not to Nutlin-3, 63 out of 100 (63%) cases revealed a normal pattern. These cases included all the 12 cases bearing 17p deletion in the absence of detectable concomitant *TP53* mutations, and all the 14 CLL cases of the analyzed cohort bearing a 11q deletion in absence of concomitant TP53 disruption (Figure 1B,C and Additional file 2: Figure S2). Consistently, Nutlin-3 treated CLL cells from all these 63 cases displayed a high mortality in vitro by the Annexin V/7-AAD assay (not shown).

Eighteen out of 100 CLL cases (18%) displayed a mutant TP53 pattern (i.e. high basal level of TP53 without increase upon Nutlin-3 exposure; Figure 1B,C and Additional file 2: Figure S2). All these cases showed a *TP53* mutated status by direct sequencing, associated (15/18) or not with a concomitant 17p deletion (Figure 1C and Additional file 2: Figure S2). Consistently, all these cases had low mortality rates in vitro by Annexin V/7-AAD assay without differences between Nutlin-3 treated and untreated conditions (not shown).

Finally, 19 out of 100 CLL cases (19%) showed an intermediate pattern, characterized by an important basal accumulation of TP53, increased upon incubation with Nutlin-3 (Figure 1B,C and Additional file 2: Figure S2). Patients belonging to this third group presented different mortality levels by Annexin V assay (not shown). Eight out of 19 CLL cases with an intermediate pattern showed a mutated *TP53* status (3 cases with concomitant 17p deletion) whereas the remaining 11 cases did not show a mutated *TP53* status by using direct sequencing (Figure 1C and Additional file 2: Figure S2). Of note, among the 11 cases with an intermediate pattern not showing a *TP53* mutated status by direct sequencing, none was 17p deleted.

### Evaluation of TP53 target genes

No differentially expressed genes were found between Nutlin-3 treated and untreated CLL cells by utilizing a global gene expression profiling approach comparing Nutlin-3 treated versus untreated samples of 7 *TP53*^del/mut^ CLL cases (not shown). Consistently, when *TP53*^del/mut^ CLL cells, exposed or not to Nutlin-3, were tested for the modulation of the genes previously identified to represent the signature of Nutlin-3 exposed *TP53*^wt/wt^ CLL cells [32], none of these genes were found to be differentially expressed upon Nutlin-3 exposure (Additional file 2: Figure S3), including the TP53 target genes *CDKN1A*, *BAX* and *PUMA*[32].

**Figure 2:**
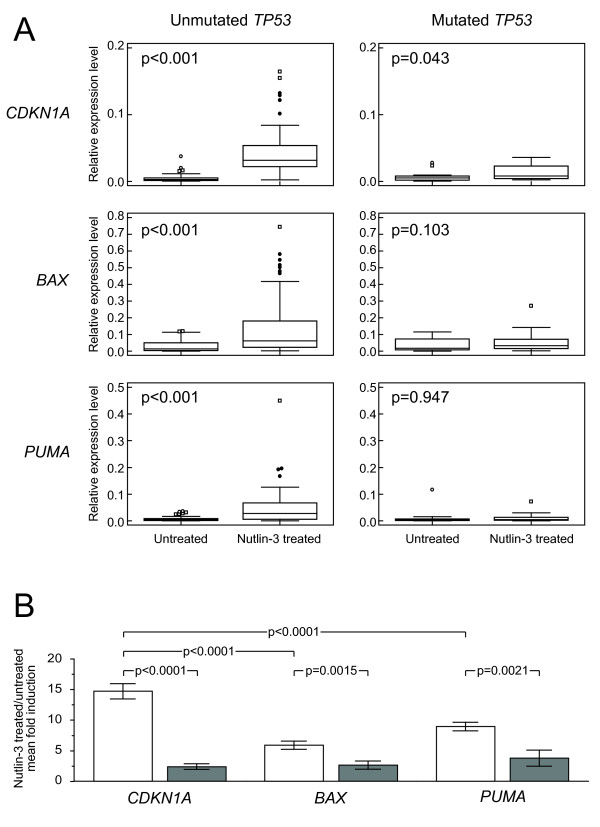
**Induction of the TP53 target genes *CDKN1A*, *BAX *and *PUMA *in CLL cases of the training cohort with a *TP53 *mutated status (*TP53***^**mut/del **^**or *TP53***^**mut/wt **^**genotypes) or a*TP53 *wild type status evaluated by direct sequencing. A)** Box-and-whisker plots show data obtained by qRT-PCR evaluation of *CDKN1A*, *BAX* and *PUMA* expression levels in untreated or Nutlin-3 treated samples of CLL cases of the training cohort with a mutated or with an unmutated *TP53* status. The corresponding p value (Student’s *t*-test) is reported. **B)** Histograms represent Nutlin-3 treated/untreated mean fold induction in *TP53* wild type (white bar) and *TP53* mutated (*TP53*^mut/del^ or *TP53*^mut/wt^ genotypes, grey bar) CLL cases. Reported p values refer to Student’s *t*-test. Error bars represent SD.

According to these results, we evaluated the expression levels of *CDKN1A*, *BAX* and *PUMA* upon Nutlin-3 treatment in CLL cases with an unmutated *TP53* status (*TP53*^wt/wt^ or *TP53*^del/wt^ genotype, overall accounting for 74 cases) versus cases with a mutated *TP53* status (*TP53*^mut/del^ or *TP53*^mut/wt^ genotype, overall accounting for 26 cases), as defined by direct sequencing. As shown in Figure 2A, CLL cases with an unmutated *TP53* status had a marked induction upon Nutlin-3 exposure for all the three genes (0.0031 versus 0.0318, *CDKN1A*; 0.0155 versus 0.0641, *BAX*; 0.0047 versus 0.0274, *PUMA*; p < 0.001 for all the comparisons). This also held true for CLL cases carrying a 11q deletion, which were characterized by a marked induction for all the three genes, not dissimilar from that of CLL cases with a normal karyotype or carrying 13q deletion or trisomy 12 (not shown). On the contrary, in CLL cases with a mutated *TP53* status, *CDKN1A* expression levels of Nutlin-3 treated and untreated samples barely reached a significant difference (0.042 versus 0.0083, p = 0.043) and, in a similar manner, failed to reach a significant difference for *BAX* and *PUMA* (0.0164 versus 0.0354, p = 0.103, *BAX*; 0.0033 versus 0.0049, p = 0.947, *PUMA*). In keeping with previous reports [26], upon Nutlin-3 treatment, *CDKN1A* expression levels showed the greatest difference of increases between cases with an unmutated and a mutated *TP53* status (14.73 vs 2.42, p < 0.0001) and the greatest amplitude of induction in cases with an unmutated *TP53* status when compared to induction of *BAX* (5.93. versus 2.68, p = 0.0015) and *PUMA* (8.98 versus 3.80, p = 0.0021). Altogether, we chose to employ *CDKN1A* as TP53 target gene in the context of an assay for the functional evaluation of *TP53* status (Figure 2B).

**Figure 3:**
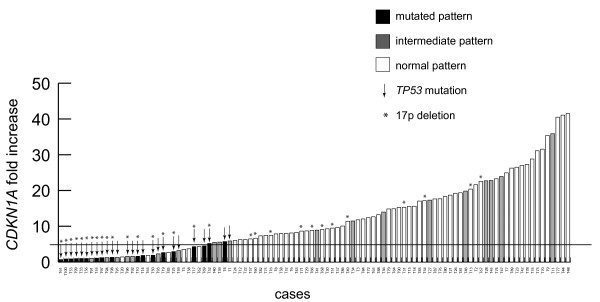
**Functional classification of CLL cases of the training cohort according to both western blot assay and evaluation of *CDKN1A *induction by qRT-PCR.** Histograms show data, obtained by qRT-PCR amplification, of *CDKN1A* fold increase expression for each CLL case of the training cohort classified according to western blot assay. Arrows indicate CLL cases with a *TP53* gene mutated status as evaluated by direct sequencing, asterisks indicate CLL cases with a 17p deletion.

As shown in Figure 3, when the whole cohort of 100 cases was plotted according to the evaluation of *CDKN1A* expression level increases, all the 26 cases with a *TP53* mutated status (*TP53*^del/mut^ or *TP53*^mut/wt^ cases) clustered in the left part of the graph by expressing *CDKN1A* levels below (23/26) or slightly above 5-fold increase (3/26). Consistently, the great majority (57/63, 90.5%) of cases with a normal pattern in vitro had *CDKN1A* values clearly above the 5-fold increase. Finally, as many as 10/12 cases with intermediate pattern in vitro without evidence of TP53 derangement by FISH and direct sequencing had a *CDKN1A* increase above the 5-fold threshold.

### Functional characterization of TP53 dysfunction in an independent validation cohort

In order to validate the functional assay here proposed, we analyzed a second independent cohort of 40 patients, purposely enriched in CLL cases with a TP53 disrupted status evaluated by FISH and direct sequencing, composed by: i) 24 cases with *TP53* mutation with concomitant 17p deletion (*TP53*^del/mut^, 12 cases) or without concomitant 17p deletion (*TP53*^mut/wt^, 12 cases); ii) 3 cases bearing a 17p deletion in absence of a concomitant *TP53* mutation (*TP53*^del/wt^); iii) 4 cases bearing a 11q deletion in absence of concomitant 17p deletion and/or *TP53* mutation; iv) 9 cases without *TP53* mutation, 17p deletion and/or 11q deletion (Additional file 1: Table S3). As shown in Additional file 2: Figure S4, for each case of this cohort, both western blotting for TP53 and qRT-PCR for *CDKN1A* upon in vitro Nutlin-3 treatment of CLL cells were performed. Results of these analyses on the validation cohort were separately evaluated “in blind” by 5 independent data analyzers. The sensitivity of the proposed functional assay resulted 0.9 (0.78-1, 95% Confidence Interval, CI), whilst the specificity resulted 0.875 (0.713-1, 95% CI). In detail, for 35 out of 40 cases the 5 analyzers concordantly defined a TP53 status in keeping with the *TP53* genotype, as evaluated by FISH and direct sequencing (Additional file 1: Table S4). Although apparently not expressing TP53 mutations by direct sequencing, two cases (e.g. V6, V16) were concordantly defined as with a dysfunction by the 5 data analyzers. Nevertheless, given the low *CDKN1A* increases found for these two cases (Additional file 2: Figure S4B), the concordant readouts of the 5 data analyzers could be considered in keeping with the documented higher sensitivity of the functional test compared with the *TP53* direct sequencing. Finally, for 3 out of 40 cases (e.g. V29, V40, V42), the TP53 pathway functionality was not correctly defined, although in 1 case (e.g. V29) 2 out of 5 data analyzers attributed a functional status consistent with the *TP53* genotype (Additional file 1: Table S4).

### Comparison with alternative TP53 functional assays

A series of 10 CLL cases composed by 4 11q deleted/*TP53*^wt/wt^ cases, 1 *TP53*^del/mut^ cases, 2 *TP53*^mut/wt^ and 3 *TP53*^wt/wt^ cases was evaluated for the presence of ATM alterations by performing alternative treatments with etoposide, etoposide plus Nutlin-3 or Nutlin-3, as previously reported [21] (Additional file 1: Table S5). The alternative treatments were set up on the 4 cell lines EHEB, MEC-1, RAJI, and HL60 (Additional file 2: Figure S5A). As shown in Additional file 2: Figure S5B, with the only exclusion of 2 out of 4 11q deleted cases, no relevant differences were detected in terms of type of response among experiments with the alternative treatments.

The same series of 10 CLL cases was also employed to perform a comparison between the approach by western blot/qRT-PCR and an alternative approach by FACS evaluating the modulation of TP53 and *CDKN1A* protein expression [26,33,34]. Again, FACS analysis was set up on the series of 4 cell lines EHEB, MEC-1, RAJI, and HL60. As shown in Additional file 2: Figure S6A, the obtained patterns were consistent with those obtained for the western blot/qRT-PCR approach and in keeping with the cell line *TP53* genotype (Additional file 2: Figure S6A). In the same manner, results of FACS analysis for evaluation of TP53 and CDKN1A protein expression on the series of 10 CLL cases did not significantly diverge from those obtained for the western blot/qRT-PCR approach here proposed (Additional file 1: Table S6 and Additional file 2: Figure S6B).

## Discussion

Impaired TP53 function through mutation and/or deletion is the most characterized factor associated with chemoresistance in CLL [15]. Currently, FISH is a widely used technique to detect chromosomal abnormalities such 17p deletion in CLL but there is not a complete overlap between 17p deletion and *TP53* mutation, although the deletion of one *TP53* allele is frequently accompanied by mutation of the other allele. Direct sequencing is considered the standard technique for the detection of *TP53* mutations, with a sensitivity of about 15%-20% of mutated DNA, but it is a time consuming test, that does not take in account TP53 functionality. In this context, a functional assessment of TP53 pathway becomes of interest in high risk CLL patient, especially in the subgroup that may escape FISH or mutational analysis due to low 17p deleted/*TP53* mutated clone size. To address this issue in the last decade several TP53 functional assays have been proposed (Additional file 1: Table S7) [20-26,28,29,34].

By applying a training/validation strategy on a cohort of 140 CLL, largely enriched for cases with a TP53 disrupted status, we compared a TP53 functional assay, based on the combined evaluation of TP53 protein expression levels by western blotting and of *CDKN1A* transcript expression levels by qRT-PCR, with the canonical evaluation of *TP53* mutational status by direct sequencing. Cases identified as with a normal pattern by western blot, and with high induction of *CDKN1A* expression level, consistently presented an unmutated *TP53* status by direct sequencing, also irrespective of the presence of a 17p deletion (see for example T13, T15, T51, T71 in Additional file 2: Figure S2). Of note, this type of response was also shared by all the cases of the training and of the validation cohorts in which an 11q deletion was not associated with a concomitant *TP53* disrupted status. Cases identified as with a mutant pattern by western blot and with low induction of *CDKN1A* expression level were consistently *TP53* mutated by direct sequencing. Low *CDKN1A* increases characterized cases with an intermediate pattern in which *TP53* mutations were detectable by direct sequencing. On the contrary, cases with an intermediate pattern and with high induction of *CDKN1A* expression level were consistently *TP53* unmutated by direct sequencing. Since there is a tight correlation between the size of the *TP53* mutant clone and the impaired response to evaluation of TP53 functionality, the latter could be cases with a small TP53 disrupted clone size, that can be unveiled by western blot analysis but not by qRT-PCR where the lack of *CDKN1A* induction by the *TP53* mutated sub-clone is overcome by its up regulation occurring in the normal *TP53* component. Finally, cases with a normal pattern by western blot but low *CDKN1A* increases (i.e. T20, T63, T85 in Additional file 2: Figure S2) could be characterized by defects on DNA damage pathway other than TP53 defects such as type C defects [23].

The assay here proposed, being based on the use of Nutlin-3 as TP53 activator, is not specifically focused on the detection of ATM mutation as they are other assays based on the combinatorial use of etoposide plus Nutlin-3 [21]. In our comparison series, in 2 out of 4 11q deleted cases, differences were detected in terms of type of response between experiments carried out by exposing CLL cells to Nutlin-3 and experiments performed by utilizing the combinatorial strategy (Additional file 2: Figure S5). In this context, a lack of the revelation of a dysfunctional response in the case of for 11q deleted cases could depend on the residual function of the remaining allele [29,35], although ATM sequencing was not performed in this study and, therefore, a loss of heterozygosis was not specifically investigated in these cases. This could be viewed as a limit of the proposed western blot/qRT-PCR approach although, from a clinical point of view, ATM defects, associated with a failure of TP53 activation, are no longer to be considered a high risk category [35]. These aspects could indicate different mechanisms for TP53 activation between etoposide or irradiation and alkylating agents or purine analogs [35]. Also in the light of the consideration mentioned above, we chose to employ a relatively pure TP53 activator such as Nutlin-3, given its non-genotoxic features that simplify its handling.

Differently from the TP53 functional assay here proposed, an approach by using FACS could be applied to samples with low tumor load without a previous step of purification of the neoplastic component [26,33,34]. On the other hand, a downside of the FACS approach could be the experimental variability due to the efficiency of the permeabilization procedure and intra-cytoplasmic staining of TP53 and CDKN1A proteins. In fact, in the comparison with western blotting, the FACS methodology showed lower signals, especially for the evaluation of TP53 expression levels (Additional file 2: Figure S6). Therefore, evaluation of TP53 expression levels by western blot seems in our hands a more powerful approach, particularly useful in cases with low TP53 dysfunctional sub-clones (Additional file 2: Figure S6). In addition, the FACS approach does not allow to highlight TP53 frameshift mutations that are, instead, easily defined by western blot (Figure 1, Additional file 2: Figure S2, Figure S5 and Figure S6).

**Figure 4:**
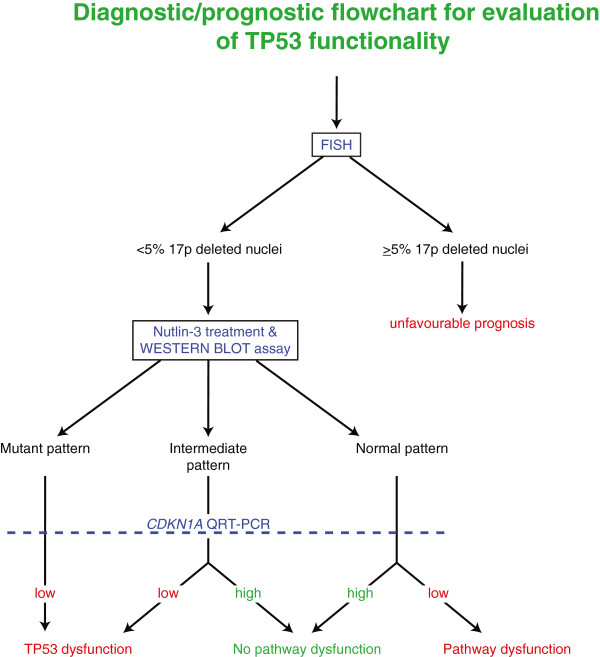
Diagnostic/prognostic flowchart for evaluation of TP53 functionality of CLL cases.

According to the above mentioned results, we propose a diagnostic flowchart based on the investigation for 17p deletion by FISH and the evaluation of TP53 pathway functionality by the assay combining western blotting for TP53 and qRT-PCR for *CDKN1A* upon a 24-hour in-vitro incubation of CLL cells with the non-genotoxic TP53 activator Nutlin-3. As shown in Figure 4, the proposed functional assay might be particularly useful in CLL cases in which a significant amount of 17p deleted nuclei are not detected by the conventional FISH analysis. In particular, the proposed functional assay is able to identify cases with dysfunctional responses to TP53 activators when a mutant pattern (always associated with a reduced up regulation of *CDKN1A*) or an intermediate pattern by western blot is documented, or when a normal/intermediate western blot pattern is associated with a reduced *CDKN1A* up regulation. In this context, the higher sensitivity of western blot if compared with direct sequencing might make the proposed assay of potential utility as a tool for the detection of chemoresistant cases bearing dysfunctional TP53 at a sub-clonal level. Clinically, the capability to detect TP53 dysfunctional sub-clones with small cell size (i.e. <15-20% of leukemic cell population) could be very important at diagnosis or at early disease phases to anticipate disease aspects such as chemorefractoriness and relapse, as well as to detect a minimal residual clone (Additional file 1: Table S7). However, further studies are needed to collect definitive information on the clinical relevance of TP53 dysfunction, as evaluated by the western blot/qRT-PCR assay in the proposed diagnostic flowchart, in comparison with the conventional and widely adopted combination of FISH analysis and *TP53* direct sequencing [2-4,7,8,18-29]. Finally, it is noteworthy that the assay here proposed, differently from the conventional FISH analysis/*TP53* direct sequencing approach, by evaluating *CDKN1A* expression levels by qRT-PCR, could be of relevant utility to define cases with a type C defect that has been associated both with short progression free survival intervals and with early relapse [35].

## Conclusions

The functional assay here proposed has the advantage of a relatively low cost, as compared to direct sequencing, and might contribute: i) to define dysfunctional cases that may escape FISH and *TP53* mutational analysis by direct sequencing due to low 17p deleted/*TP53* mutated clone size; ii) to define dysfunctional cases not exhibiting 17p deletion or *TP53* mutations (e.g. dysfunctional cases with a type C defect). The flowchart here proposed could represent a valuable contribution to diagnostic and prognostic criteria for CLL patients, especially for those affected by a high risk disease form.

## Methods

### Patients and cell lines

This study was performed on a multi-institutional cohort of 100 CLL utilized to set-up the flow chart for TP53 status evaluation in CLL (training cohort), and a second multi-institutional independent cohort of 40 CLL, employed to validate the TP53 status flowchart utilizing a “blind” approach (validation cohort), diagnosed according to the IWCLL-NCI criteria [36]. All patients were provided informed consent in accordance with local Institution Review Board requirements and the Declaration of Helsinki. The main clinical and biological features of the two cohorts are summarized in Additional file 1: Table S2 (training cohort) and in Additional file 1: Table S3 (validation cohort). In this context, in 123/140 cases the neoplastic component was more than 80% of cells; in the remaining 17 cases the in vitro experiments (see below) were performed upon B cell purification by CD19^+^ conjugated columns (Miltenyi Biotec). Sequences of *IGHV* genes were performed as previously reported, using the ImMunoGeneTics (IMGT) directory for the identification of *IGHV-D-J* rearrangements and a 2% cut off of for mutated/unmutated discrimination [37]. Detection of CD38, CD49d and ZAP70 expression was performed as previously reported using the cut offs of 30% positive cells (CD38, CD49d expression) or 20% of positive cells (ZAP70 expression) [37].

EHEB, MEC-1, RAJI and HL-60 cell lines were obtained from DSMZ (Germany); MAVER-1 cell line was kindly provided by Alberto Zamò (Department of Pathology, University of Verona, Italy). The most relevant molecular features of cell lines to address the study are summarized in Additional file 1: Table S1.

### Analysis of cytogenetic aberrations

Cytogenetic abnormalities were detected as previously reported [37] by FISH for deletion on chromosomes 11, 13, and 17 using the 3 locus specific probes LSI-ATM (SpectrumGreen), LSI-D13S319 (SpectrumOrange), and LSI-p53 (SpectrumOrange), and for aneuploidy of chromosome 12 using an alpha satellite DNA probe CEP12 (SpectrumGreen) (Vysis). In all cases, at least 200 interphase cells with well delineated fluorescent spots were examined. A cut-off of 5% of nuclei was applied to discriminate between negative cases and cases bearing a specific chromosomal abnormality [37]. In the case of 17p deletion, all cases with deletion detectable in 5-10% of nuclei were re-evaluated with a second different specific LSI-p53 probe (Metasystems).

### Analysis of *TP53* mutations

In CLL cases and cell lines, mutation analysis of *TP53* exons 2 to 11 was done by DNA direct sequencing on an ABI Prism 3130 automated DNA sequence analyzer (Applied Biosystems) according to the IARC guidelines (http://www.p53.iarc.fr) and analyzed by Sequencing Analysis v5.2 software (Applied Biosystems) [7]. Mutations were confirmed on both strands on independent amplimers and validated by the IARC *TP53* Mutation Database R15 [31].

### Cell culture conditions

Primary CLL were obtained from peripheral blood samples by Ficoll-Hypaque (Pharmacia) density gradient centrifugation and cryopreserved until use. After thawing and upon evaluation of the percentage of CLL cells by flow cytometry, CLL cells were further purified by immunomagnetic negative selection as described [38].

Cell lines and CLL cells were cultured (1 × 10^7^ cells/ml) in RPMI-1640 (Biochrom) supplemented with 10% heat-inactivated fetal bovine serum (Biochrom), 100 U/ml penicillin, 0.1 mg/ml streptomycin and 2 mM L-glutamine (Invitrogen) in the presence or not of 10 μM Nutlin-3 (Cayman Chemical) or 50 μM etoposide, or 50 μM etoposide plus 10 μM Nutlin-3 for 24 hours (CLL cases) [21,32], or up to 48 hours for cell lines in case of Nutlin-3 treatment, as previously reported [32]. Cell viability was assayed by AnnexinV and 7-amino-actinomycin-D (7-AAD, both from Becton-Dickinson) double staining (AnnexinV/7-AAD assay) and data were acquired on a FACSCanto flow cytometer and analyzed by Diva software (Becton-Dickinson) as previously reported [38].

### Western blot

Total proteins were extracted in RIPA lysis buffer (Santa Cruz) from cultured cells, collected 24 hours after Nutlin-3 treatment, quantified with Bradford assay (Bio-Rad) and ran in 10% SDS-PAGE gels prior to transfer to nitrocellulose membranes (GE Healtcare) for immunoblot analysis and detection by ECL (GE Healtcare) or Immobilon (Millipore Corporation). 1:1000 mouse-anti-TP53 (clone DO1, sc-126) was used for protein detection (Santa Cruz). 1:100.000 mouse-anti-β-actin antibody (clone AC-74, Sigma-Aldrich) was used as loading control.

### TP53 and CDKN1A flow cytometry

Fixation and permeabilization of cells were performed with Fix and Perm (Caltag) with methanol modification according to producer guidelines. Cell staining was performed using direct anti-p53(clone DO7)-FITC (sc-47698, Santa Cruz) or indirect anti-p21 (clone Ab-1, OP64, Calbiochem) with secondary antimouse IgG-FITC (sc-2010, Santa Cruz) or anti-CD19-APC (clone HIB19, 555415, BD-Pharmingen) or specific isotype controls. Data were acquired on a FACSCanto flow cytometer and analyzed by Diva software (Becton-Dickinson) as previously reported [38].

### Gene expression profile experiments

Gene expression profile experiments (GEP) were performed in a cohort of 20 CLL cases (Additional file 1: Table S8). Total RNA was extracted from purified CLL cells and normal peripheral blood B cells of healthy donors using Trizol reagent (Invitrogen) and validated for integrity and purity using the Agilent 2100 Bioanalyzer (Agilent Tecnologies). GEP was performed using the whole-human genome (4×44K) oligo microarray platform (Agilent Tecnologies) as previously described [39]. Microarray slides were analyzed as previously described [39]. Bioinformatic analyses were performed using SAM algorithm.

### Quantitative real-time PCR (qRT-PCR)

Expression of specific genes of interest (i.e. *CDKN1A*, *BAX*, *PUMA* and *B2M*) was evaluated with the TaqMan Gene Expression assay kit (Applied Biosystem, Life Tecnologies); the relative amount of each gene was calculated utilizing the expression of *B2M* as internal control using the equation 2^-ΔCt^ where ΔCt = (Ct_gene_-Ct_*B2M*_). Fold changes between classes were calculated as reported [39]. All qRT-PCR experiments were performed on an Applied Biosystem 7700 Sequence Detection System (Applied Biosystem).

### Statistical analysis

Data were compared using Student’s *t*-test for independent or paired samples. All statistical analyses were performed using the MedCalc software (MedCalc Software).

## Abbreviations

FISH: Fluorescence in situ hybridization; qRT-PCR: Quantitative real time PCR; FACS: Fluorescence activated cell sorting; GEP: Gene expression profiling; 7-AAD: 7-amino-actinomycin.

## Competing interests

The authors declare that they have no competing interests.

## Authors’ contribution

FP contribute to write the manuscript, analyzed the data and performed the research, MDB wrote the manuscript and analyzed the data, NP, RB, AZ, FMR, MD, PS contributed to perform the research, AC, AG, FDR, FZ, GP, DR, GG, GDP, GZ, RF, AG provided well characterized biological samples and contributed to write the manuscript, VG designed the study and contributed to write the manuscript. All authors read and approved the final manuscript.

## Supplementary Material

Additional file 1 **Additional tables: Table S1. ***TP53* mutational status of the cell lines employed in the study. **Table S2.** Characterization of CLL cases of the training cohort. **Table S3.** Characterization of CLL cases of the validation cohort. **Table S4.** Validation cohort: evaluation “in blind” of 5 independent data analyzers. **Table S5.** Characterization of CLL cases used for comparison experiments. **Table S6.** Median fluorescence intensity values for TP53 and CDKN1A protein expression by FACS analysis. **Table S7.** Overview of the peculiar features of the different TP53 functional assays described by literature. **Table S8.** Characterization of CLL cases used for microarray experiments. Click here for file

Additional file 2 **Additional figures: Figure S1.** Evaluation of apoptosis levels by Annexin V-7AAD and sensitivity of western blot analysis upon Nutlin-3 treatment. **Figure S2.** Western blot for CLL cases of the training cohort. **Figure S3.** Lack of the gene expression signature associated with Nutlin-3 treatment in *TP53*^del/mut^ CLL samples. **Figure S4.** Western blot/qRT PCR assay for CLL cases of the validation cohort. **Figure S5.** Comparison with alternative TP53 functional assays using combinatorial strategies with etoposide. **Figure S6.** Comparison with evaluation of TP53 and CDKN1A protein expression levels by FACS analysis. Click here for file

## References

[B1] Chiorazzi N Rai KR Ferrarini M Chronic Lymphocytic Leukemia N Engl J Med 2005 352 804 815 10.1056/NEJMra041720 15728813

[B2] Catovsky D Richards S Matutes E Oscier D Dyer MJ Bezares RF Pettitt AR Hamblin T Milligan DW Child JA Hamilton MS Dearden CE Smith AG Bosanquet AG Davis Z Brito-Babapulle V Else M Wade R Hillmen P Assessment of fludarabine plus cyclophosphamide for patients with chronic lymphocytic leukaemia (the LRF CLL4 Trial): a randomised controlled trial Lancet 2007 370 230 239 10.1016/S0140-6736(07)61125-8 17658394

[B3] Dohner H Fischer K Bentz M Hansen K Benner A Cabot G Diehl D Schlenk R Coy J Stilgenbauer S p53 gene deletion predicts for poor survival and non-response to therapy with purine analogs in chronic B-cell leukemias Blood 1995 85 1580 1589 7888675

[B4] Grever MR Lucas DM Dewald GW Neuberg DS Reed JC Kitada S Flinn IW Tallman MS Appelbaum FR Larson RA Paietta E Jelinek DF Gribben JG Byrd JC Comprehensive assessment of genetic and molecular features predicting outcome in patients with chronic lymphocytic leukemia: results from the US Intergroup Phase III Trial E2997 J Clin Oncol 2007 25 799 804 10.1200/JCO.2006.08.3089 17283363

[B5] Dicker F Herholz H Schnittger S Nakao A Patten N Wu L Kern W Haferlach T Haferlach C The detection of TP53 mutations in chronic lymphocytic leukemia independently predicts rapid disease progression and is highly correlated with a complex aberrant karyotype Leukemia 2009 23 117 124 10.1038/leu.2008.274 18843282

[B6] Gonzalez D Martinez P Wade R Hockley S Oscier D Matutes E Dearden CE Richards SM Catovsky D Morgan GJ Mutational status of the TP53 gene as a predictor of response and survival in patients with chronic lymphocytic leukemia: results from the LRF CLL4 trial J Clin Oncol 2011 29 2223 2229 10.1200/JCO.2010.32.0838 21483000

[B7] Rossi D Cerri M Deambrogi C Sozzi E Cresta S Rasi S De PL Spina V Gattei V Capello D Forconi F Lauria F Gaidano G The prognostic value of TP53 mutations in chronic lymphocytic leukemia is independent of Del17p13: implications for overall survival and chemorefractoriness Clin Cancer Res 2009 15 995 1004 10.1158/1078-0432.CCR-08-1630 19188171

[B8] Zenz T Krober A Scherer K Habe S Buhler A Benner A Denzel T Winkler D Edelmann J Schwanen C Dohner H Stilgenbauer S Monoallelic TP53 inactivation is associated with poor prognosis in chronic lymphocytic leukemia: results from a detailed genetic characterization with long-term follow-up Blood 2008 112 3322 3329 10.1182/blood-2008-04-154070 18689542

[B9] Zenz T Eichhorst B Busch R Denzel T Habe S Winkler D Buhler A Edelmann J Bergmann M Hopfinger G Hensel M Hallek M Dohner H Stilgenbauer S TP53 mutation and survival in chronic lymphocytic leukemia J Clin Oncol 2010 28 4473 4479 10.1200/JCO.2009.27.8762 20697090

[B10] Vogelstein B Lane D Levine AJ Surfing the p53 network Nature 2000 408 307 310 10.1038/35042675 11099028

[B11] Clarke AR Purdie CA Harrison DJ Morris RG Bird CC Hooper ML Wyllie AH Thymocyte apoptosis induced by p53-dependent and independent pathways Nature 1993 362 849 852 10.1038/362849a0 8479523

[B12] Lane DP Cancer. p53, guardian of the genome Nature 1992 358 15 16 10.1038/358015a0 1614522

[B13] Lotem J Sachs L Hematopoietic cells from mice deficient in wild-type p53 are more resistant to induction of apoptosis by some agents Blood 1993 82 1092 1096 8353276

[B14] Lowe SW Ruley HE Jacks T Housman DE p53-dependent apoptosis modulates the cytotoxicity of anticancer agents Cell 1993 74 957 967 10.1016/0092-8674(93)90719-7 8402885

[B15] Zenz T Benner A Dohner H Stilgenbauer S Chronic lymphocytic leukemia and treatment resistance in cancer: the role of the p53 pathway Cell Cycle 2008 7 3810 3814 10.4161/cc.7.24.7245 19098429

[B16] Cang S Mukhi N Wang K Liu D Novel CD20 monoclonal antibodies for lymphoma therapy J Hematol Oncol 2012 5 64 10.1186/1756-8722-5-64 23057966 PMC3479003

[B17] Lu K Wang X Therapeutic advancement of chronic lymphocytic leukemia J Hematol Oncol 2012 5 55 10.1186/1756-8722-5-55 22980425 PMC3465197

[B18] Dohner H Stilgenbauer S Benner A Leupolt E Krober A Bullinger L Dohner K Bentz M Lichter P Genomic aberrations and survival in chronic lymphocytic leukemia N Engl J Med 2000 343 1910 1916 10.1056/NEJM200012283432602 11136261

[B19] Lozanski G Heerema NA Flinn IW Smith L Harbison J Webb J Moran M Lucas M Lin T Hackbarth ML Proffitt JH Lucas D Grever MR Byrd JC Alemtuzumab is an effective therapy for chronic lymphocytic leukemia with p53 mutations and deletions Blood 2004 103 3278 3281 10.1182/blood-2003-10-3729 14726385

[B20] Pospisilova S Gonzalez D Malcikova J Trbusek M Rossi D Kater AP Cymbalista F Eichhorst B Hallek M Dohner H Hillmen P van OM Gribben J Ghia P Montserrat E Stilgenbauer S Zenz T ERIC recommendations on TP53 mutation analysis in chronic lymphocytic leukemia Leukemia 2012 26 1458 1461 10.1038/leu.2012.25 22297721

[B21] Best OG Gardiner AC Majid A Walewska R Austen B Skowronska A Ibbotson R Stankovic T Dyer MJ Oscier DG A novel functional assay using etoposide plus nutlin-3a detects and distinguishes between ATM and TP53 mutations in CLL Leukemia 2008 22 1456 1459 10.1038/sj.leu.2405092 18200038

[B22] Chiaretti S Tavolaro S Marinelli M Messina M Del GI Mauro FR Santangelo S Piciocchi A Peragine N Truong S Patten N Ghia EM Torrente I De Propris MS Nanni M Lawrence J Guarini A Foa R Evaluation of TP53 mutations with the AmpliChip p53 research test in chronic lymphocytic leukemia: Correlation with clinical outcome and gene expression profiling Genes Chromosomes.Cancer 2011 50 263 274 21319261 10.1002/gcc.20852

[B23] Johnson GG Sherrington PD Carter A Lin K Liloglou T Field JK Pettitt AR A novel type of p53 pathway dysfunction in chronic lymphocytic leukemia resulting from two interacting single nucleotide polymorphisms within the p21 gene Cancer Res 2009 69 5210 5217 10.1158/0008-5472.CAN-09-0627 19491257

[B24] Kringen P Bergamaschi A Due EU Wang Y Tagliabue E Nesland JM Nehman A Tonisson N Borresen-Dale AL Evaluation of arrayed primer extension for TP53 mutation detection in breast and ovarian carcinomas Biotechniques 2005 39 755 761 10.2144/000112000 16312222

[B25] Mackus WJ Kater AP Grummels A Evers LM Hooijbrink B Kramer MH Castro JE Kipps TJ van Lier RA van Oers MH Eldering E Chronic lymphocytic leukemia cells display p53-dependent drug-induced Puma upregulation Leukemia 2005 19 427 434 10.1038/sj.leu.2403623 15674362

[B26] Mohr J Helfrich H Fuge M Eldering E Buhler A Winkler D Volden M Kater AP Mertens D Te RD Dohner H Stilgenbauer S Zenz T DNA damage-induced transcriptional program in CLL: biological and diagnostic implications for functional p53 testing Blood 2011 117 1622 1632 10.1182/blood-2010-08-300160 21115975

[B27] Marinelli M Peragine N Di MV Chiaretti S De Propris MS Raponi S Tavolaro S Mauro FR Del GI Guarini A Foa R Identification of molecular and functional patterns of p53 alterations in chronic lymphocytic leukemia patients in different phases of the disease Haematologica 2013 98 371 375 10.3324/haematol.2012.069906 22983585 PMC3659928

[B28] Pettitt AR Sherrington PD Stewart G Cawley JC Taylor AM Stankovic T p53 dysfunction in B-cell chronic lymphocytic leukemia: inactivation of ATM as an alternative to TP53 mutation Blood 2001 98 814 822 10.1182/blood.V98.3.814 11468183

[B29] Te Raa GD Malcikova J Pospisilova S Trbusek M Mraz M Le Garff-Tavernier M Merle-Beral H Lin K Pettitt AR Merkel O Stankovic T van Oers MH Eldering E Stilgenbauer S Zenz T Kater AP Overview of available p53 function tests in relation to TP53 and ATM gene alterations and chemoresistance in chronic lymphocytic leukemia 2013 Leuk: Lymphoma 10.3109/10428194.2013.796058 23614766

[B30] Saha MN Qiu L Chang H Targeting p53 by small molecules in hematological malignancies J Hematol Oncol 2013 6 23 10.1186/1756-8722-6-23 23531342 PMC3614876

[B31] Petitjean A Mathe E Kato S Ishioka C Tavtigian SV Hainaut P Olivier M Impact of mutant p53 functional properties on TP53 mutation patterns and tumor phenotype: lessons from recent developments in the IARC TP53 database Hum Mutat 2007 28 622 629 10.1002/humu.20495 17311302

[B32] Zauli G di Iasio MG Secchiero P Dal Bo M Marconi D Bomben R Del Poeta G Gattei V Exposure of B cell chronic lymphocytic leukemia (B-CLL) cells to nutlin-3 induces a characteristic gene expression profile, which correlates with nutlin-3-mediated cytotoxicity Curr Cancer Drug Targets 2009 9 510 518 10.2174/156800909788486777 19519319

[B33] Carter A Lin K Sherrington PD Pettitt AR Detection of p53 dysfunction by flow cytometry in chronic lymphocytic leukaemia Br J Haematol 2004 127 425 428 10.1111/j.1365-2141.2004.05223.x 15521919

[B34] Le Garff-Tavernier M Blons H Nguyen-Khac F Pannetier M Brissard M Gueguen S Jacob F Ysebaert L Susin SA Merle-Beral H Functional assessment of p53 in chronic lymphocytic leukemia Blood Cancer J 2011 1 e5 10.1038/bcj.2011.3 22829111 PMC3255272

[B35] Lin K Adamson J Johnson GG Carter A Oates M Wade R Richards S Gonzalez D Matutes E Dearden C Oscier DG Catovsky D Pettitt AR Functional analysis of the ATM-p53-p21 pathway in the LRF CLL4 trial: blockade at the level of p21 is associated with short response duration Clin Cancer Res 2012 18 4191 4200 10.1158/1078-0432.CCR-11-2936 22675167

[B36] Hallek M Cheson BD Catovsky D Caligaris-Cappio F Dighiero G Dohner H Hillmen P Keating MJ Montserrat E Rai KR Kipps TJ Guidelines for the diagnosis and treatment of chronic lymphocytic leukemia: a report from the International Workshop on Chronic Lymphocytic Leukemia updating the National Cancer Institute-Working Group 1996 guidelines Blood 2008 111 5446 5456 10.1182/blood-2007-06-093906 18216293 PMC2972576

[B37] Gattei V Bulian P Del Principe MI Zucchetto A Maurillo L Buccisano F Bomben R Dal-Bo M Luciano F Rossi FM Degan M Amadori S Del PG Relevance of CD49d protein expression as overall survival and progressive disease prognosticator in chronic lymphocytic leukemia Blood 2008 111 865 873 10.1182/blood-2007-05-092486 17959854

[B38] Zucchetto A Vaisitti T Benedetti D Tissino E Bertagnolo V Rossi D Bomben R Dal Bo M Del Principe MI Gorgone A Pozzato G Gaidano G Del PG Malavasi F Deaglio S Gattei V The CD49d/CD29 complex is physically and functionally associated with CD38 in B-cell chronic lymphocytic leukemia cells Leukemia 2012 26 1301 1312 10.1038/leu.2011.369 22289918

[B39] Bomben R Dal-Bo M Benedetti D Capello D Forconi F Marconi D Bertoni F Maffei R Laurenti L Rossi D Del Principe MI Luciano F Sozzi E Cattarossi I Zucchetto A Rossi FM Bulian P Zucca E Nicoloso MS Degan M Marasca R Efremov DG Del PG Gaidano G Gattei V Expression of mutated IGHV3-23 genes in chronic lymphocytic leukemia identifies a disease subset with peculiar clinical and biological features Clin Cancer Res 2010 16 620 628 10.1158/1078-0432.CCR-09-1638 20068100

