# Supplementary material for: Detection of TP53 dysfunction in chronic lymphocytic leukemia by an in vitro functional assay based on TP53 activation by the non-genotoxic drug Nutlin-3: a proposal for clinical application
Source: J Hematol Oncol. 2013 Nov 5;6:83. doi: 10.1186/1756-8722-6-83 (PMC4222122; doi:10.1186/1756-8722-6-83)
Supplement: Additional file 1 — Additional tables: Table S1. TP53 mutational status of the cell lines employed in the study. Table S2. Characterization of CLL cases of the training cohort. Table S3. Characterization of CLL cases of the validation cohort. Table S4. Validation cohort: evaluation “in blind” of 5 independent data analyzers. Table S5. Characterization of CLL cases used for comparison experiments. Table S6. Median fluorescence intensity values for TP53 and CDKN1A protein expression by FACS analysis. Table S7. Overview of the peculiar features of the different TP53 functional assays described by literature. Table S8. Characterization of CLL cases used for microarray experiments. [file 1756-8722-6-83-S1.pdf]

Table S1. *TP53* mutational status of the cell lines employed in the study

Cell Line	Source	*TP53* genotype	Mutation	Exon	Allelic Status	del17p
EHEB	Human chronic B cell leukemia	wt/wt				Neg
RAJI	Human B cell Burkitt lymphoma	mut/wt<br>mut/wt	c.638G>A p.R213Q<br>c.700T>C p.Y234H	6<br>7	Heterozygous<br>Heterozygous	Neg
MEC-1	Human chronic B cell leukemia	mut/del	c.949_950insC p.Q317fs*29	9	Heterozygous	Pos (60%)
MAVER-1	Human mantle cell lymphoma	mut/del	c.843C>G p.D281E	8	Homozygous	Pos (90%)
HL-60	Human acute promyelocytic leukemia	del/del				Pos(100%)

Percentage of FISH positive nuclei are reported within parentheses.

Table S2: Characterization of CLL cases of the training cohort.

ID	Mutation	Exon	Del13q	Tris12	Del11q	Del17p	IGHV status	CD38	CD49d	ZAP70
T1	c.742C>T p.R248P	7	pos (45%)	neg	pos (55%)	neg	UM	pos	pos	n.d.
T2			pos (80%)	neg	pos (20%)	neg	M	neg	neg	n.d.
T3			pos (80%)	neg	neg	neg	UM	neg	pos	pos
T4	c.797G>T p.G266V	8	pos (8%)	neg	neg	neg	M	neg	neg	pos
T5			neg	pos (60%)	neg	neg	UM	pos	pos	pos
T6			pos (40%)	neg	neg	neg	M	neg	neg	pos
T7			pos (85%)	neg	neg	neg	M	neg	neg	neg
T8			neg	neg	neg	neg	UM	pos	pos	pos
T9			neg	neg	neg	neg	UM	pos	pos	neg
T10	c.517G>A p.V173M	5	neg	neg	neg	pos (80%)	UM	pos	pos	pos
T11			neg	neg	neg	neg	UM	neg	neg	neg
T12			neg	neg	pos (60%)	neg	UM	pos	neg	neg
T13			pos (81%)	neg	neg	pos (10%)	n.d.	neg	neg	pos
T14	c.701A>C p.Y234S	7	neg	neg	neg	neg	UM	pos	pos	n.d.
T15			pos (90%)	neg	neg	pos (15%)	n.d.	neg	neg	pos
T16			neg	neg	neg	pos (70%)	UM	pos	pos	pos
T17	c.584T>C p.I195T	6	pos (80%)	neg	neg	pos (82%)	M	neg	neg	pos
T18			pos (71%)	neg	neg	neg	M	neg	pos	pos
T19	c.724T>C p.C242R	7	neg	pos (5%)	neg	pos (14%)	UM	neg	pos	pos
T20			pos (17%)	neg	neg	neg	M	neg	neg	pos
T21			neg	pos (67%)	neg	neg	UM	neg	pos	neg
T22			pos (76%)	neg	neg	neg	M	neg	neg	pos
T23			neg	pos (35%)	neg	neg	UM	pos	pos	pos
T24			neg	neg	pos (6%)	pos (5%)	M	neg	pos	pos
T25	c.818G>A p.R273H	8	pos (55%)	neg	neg	pos (53%)	UM	pos	neg	pos
T26			neg	neg	neg	neg	M	neg	neg	pos
T27			neg	neg	neg	neg	UM	neg	pos	pos
T28			pos (84%)	neg	neg	neg	M	neg	neg	neg
T29			neg	neg	neg	neg	n.d.	pos	pos	pos
T30			pos (38%)	neg	neg	neg	M	neg	neg	neg
T31			neg	pos (72%)	neg	neg	UM	neg	pos	neg
T32			pos (32%)	neg	neg	neg	M	neg	neg	neg
T33	c.97-2A>T	i3	pos (61%)	neg	neg	pos (38%)	M	neg	neg	pos
T34			neg	neg	neg	neg	M	neg	neg	pos
T35			pos (55%)	neg	pos (5%)	neg	UM	pos	neg	pos
T36			pos (15%)	pos (71%)	neg	neg	Biclon. UM	pos	pos	pos
T37			neg	neg	neg	neg	UM	pos	pos	pos
T38			pos (50%)	neg	neg	neg	M	neg	neg	neg
T39			pos (15%)	neg	neg	neg	M	neg	pos	neg
T40			pos (70%)	neg	neg	neg	Biclon. M	neg	neg	neg
T41			neg	pos (70%)	neg	neg	M	neg	pos	neg
T42			pos (71%)	neg	neg	neg	M	neg	neg	neg
T43			pos (15%)	neg	neg	neg	M	neg	neg	neg
T44			neg	neg	neg	neg	UM	pos	neg	neg
T45			neg	neg	neg	neg	M	pos	neg	neg
T46			pos (92%)	neg	neg	neg	M	neg	neg	neg
T47			neg	neg	neg	neg	M	neg	neg	n.d.
T48			neg	neg	pos (83%)	neg	UM	neg	neg	pos
T49			pos (60%)	neg	pos (60%)	pos (10%)	UM	pos	pos	pos
T50	c.578A>T p.H193L	6	pos (95%)	neg	neg	pos (90%)	n.d.	neg	neg	pos
T51			pos (32%)	neg	neg	pos (18%)	n.d.	neg	neg	n.d.
T52	c.713G>T p.C238F	7	pos (85%)	neg	pos (65%)	pos (40%)	M	neg	neg	pos
T53	c.814G>T p.V272L	8	neg	neg	neg	pos (70%)	M	neg	pos	neg
T54			neg	neg	pos (80%)	pos (9%)	UM	pos	pos	neg
T55	c.824G>T p.C275F	8	neg	neg	neg	pos (60%)	M	neg	pos	n.d.
T56	c.845G>C p.R282P	8	neg	neg	neg	pos (30%)	UM	neg	neg	n.d.
T57			neg	pos (30%)	neg	pos (10%)	UM	pos	neg	pos
T58			neg	pos (70%)	pos (80%)	neg	UM	pos	pos	n.d.
T59	c.484A>T p.I162F	5	neg	neg	neg	neg	UM	neg	pos	n.d.
	c.745A>T p.R249W	7								
T60			pos (70%)	neg	neg	neg	UM	neg	neg	pos
T61	c.818G>A p.R273H	8	neg	pos (25%)	neg	pos (18%)	UM	neg	neg	neg
T62			neg	pos (61%)	neg	neg	UM	pos	pos	neg
T63			pos (65%)	neg	neg	neg	M	pos	pos	neg
T64			pos (12%)	neg	pos (72%)	neg	Biclon. UM	pos	pos	pos
T65			pos (89%)	pos (15%)	pos (80%)	neg	UM	neg	neg	pos
T66			pos (52%)	neg	neg	pos (6%)	M	neg	neg	pos
T67			neg	neg	pos (10%)	pos (6%)	UM	neg	neg	pos
T68			pos (87%)	neg	neg	pos (8%)	M	neg	neg	pos
T69	c.626_627delGA p.R209Kfs*6	6	pos (90%)	neg	pos (15%)	neg	M	neg	neg	pos
T70			neg	neg	pos (80%)	neg	nd	neg	neg	n.d.
T71			pos (75%)	neg	neg	pos (61%)	UM	neg	pos	neg

T72			neg	pos (80%)	pos (81%)	neg	UM	pos	pos	pos
T73			neg	pos (66%)	pos (7%)	neg	UM	pos	pos	pos
T74			neg	neg	pos (36%)	neg	UM	neg	neg	pos
T75			neg	pos (66%)	neg	neg	UM	pos	pos	pos
T76			pos (8%)	neg	pos (9%)	neg	UM	neg	neg	pos
T77			pos (5%)	neg	pos (73%)	neg	UM	pos	neg	pos
T78			pos (60%)	neg	neg	neg	UM	neg	neg	pos
T79			pos (64%)	neg	neg	neg	M	neg	neg	neg
T80			pos (72%)	neg	neg	neg	UM	pos	neg	pos
T81			pos (77%)	neg	neg	neg	n.d.	neg	neg	neg
T82			pos (74%)	neg	neg	neg	M	neg	neg	neg
T83			pos (65%)	pos (66%)	neg	neg	M	pos	pos	neg
T84			neg	neg	neg	neg	UM	pos	pos	pos
T85			pos (40%)	pos (49%)	pos (5%)	neg	UM	neg	pos	neg
T86			pos (60%)	pos (66%)	neg	neg	M	neg	pos	neg
T87			pos (49%)	neg	neg	neg	M	pos	pos	pos
T88			neg	neg	neg	neg	M	neg	neg	neg
T89			neg	neg	neg	neg	M	neg	pos	neg
T90			pos (64%)	neg	neg	neg	n.d.	neg	neg	neg
T91	c.989T>C p.L330P	9	pos (6%)	neg	neg	pos (81%)	UM	neg	n.d.	neg
T92	c.818G>A p.R273H	8	pos (95%)	neg	neg	neg	M	neg	n.d.	neg
T93	c.742C>T p.R248W	7	neg	neg	neg	neg	UM	pos	n.d.	neg
T94	c.824G>A p.C275Y	8	neg	pos (70%)	neg	pos (73%)	UM	pos	n.d.	neg
T95	c.847C>T p.A283C	8	pos (32%)	neg	neg	neg	M	neg	n.d.	neg
T96	c.818G>A p.A273H	8	pos (90%)	neg	neg	pos (50%)	M	neg	n.d.	neg
T97	c.338T>C p.F113S	4	pos (6%)	neg	neg	pos (5%)	UM	pos	n.d.	pos
T98	c.961A>T p.K321*	9	neg	neg	pos (5%)	pos (6%)	M	n.d.	n.d.	n.d.
T99	c.722C>T p.S241F	7	neg	neg	pos (57%)	pos (96%)	UM	pos	n.d.	neg
T100	c.378C>G p.Y126*	5	neg	neg	neg	pos (95%)	UM	neg	n.d.	pos

pos: positive; neg: negative; biclon: biclonal; M: mutated; UM: unmutated; n.d.: not determined

Cut-off for CD38 and CD49d: 30% of expressing cells; cut-off for ZAP70: 20% of expressing cells; cut-off for FISH (del13q, tris12, del11q, del17p probes): 5% of deleted nuclei

Percentage of FISH positive nuclei are reported within parentheses

Table S3: Characterization of CLL cases of the validation cohort.

ID	Mutation	Exon	Tris12	Del13q	Del11q	Del17p	IGHV status	CD38	CD49d	ZAP70
V1	c.742C>T p.R248W	7	neg	pos (82%)	pos (81%)	pos (49%)	UM	pos	pos	neg
V2	c.312delG; p.G105fs*18	4	neg	neg	neg	neg	UM	neg	neg	neg
	c.673-2A>T	7								
V4		0	neg	neg	neg	neg	UM	neg	neg	neg
V5	c.731G>A p.G244D	7	neg	neg	neg	neg	UM	pos	pos	pos
	c.733G>A p.G245S	7								
V6		0	neg	pos (18%)	neg	neg	M	neg	neg	neg
V7		0	pos (62%)	neg	neg	neg	M	pos	neg	neg
V8	c.470T>G p.V157G	5	neg	neg	neg	neg	UM	neg	pos	neg
	c.785G>T p.G262V	8								
V9	c.809T>C p.F270S	8	neg	pos (75%)	neg	pos (37%)	M	neg	neg	neg
V10	c.761T>C p.I254T	7	neg	pos (63%)	neg	pos (63%)	UM	neg	neg	neg
V11		0	neg	neg	neg	neg	UM	neg	neg	neg
V12	c.687T>A p.C229*	7	neg	neg	neg	neg	UM	neg	neg	neg
V13	c.672+1G>T	6	pos (12%)	pos (62%)	neg	neg	M	n.d.	n.d.	neg
V14		0	pos (89%)	neg	pos (9%)	neg	UM	neg	pos	pos
V15		0	neg	pos (85%)	pos (81%)	neg	UM	neg	neg	neg
V16		0	pos (61%)	neg	neg	neg	M	neg	neg	neg
V17		0	neg	pos (75%)	neg	neg	M	pos	pos	neg
V18		0	neg	pos (79%)	neg	neg	M	n.d.	neg	neg
V19		0	pos (85%)	neg	pos (14%)	neg	UM	pos	pos	neg
V20	c.532delC p.H178fs*69	5	neg	pos (80%)	neg	pos (25%)	M	pos	pos	neg
	c.892G>T p.E298*	8								
V21	c.613T>G p.Y205D	6	neg	pos (72%)	neg	pos (45%)	UM	neg	pos	neg
V22	c.733G>A p.G245S	7	neg	pos (54%)	neg	pos (60%)	M	neg	neg	neg
V23	c.757A>C p.T253P	7	neg	neg	neg	pos (45%)	M	neg	pos	pos
V24	c.584T>C p.I195T	6	neg	n.d.	neg	pos (77%)	M	neg	neg	n.d.
V25		0	neg	neg	neg	pos (6%)	UM	pos	pos	pos
V26	c.742C>G p.R248G	7	pos (55%)	neg	neg	neg	UM	neg	pos	pos
V27	c.844C>T p.R282W	8	neg	pos (14%)	neg	neg	UM	pos	pos	pos
V28	c.626_627delGA p.R209fs*6	6	neg	neg	pos (12,5%)	neg	M	neg	neg	neg
V29	c.818G>A p.R273H	8	neg	n.d.	neg	neg	M	neg	neg	n.d.
V30	c.590T>A p.V197E	6	neg	neg	neg	neg	UM	pos	pos	neg
V32	c.469 G>T p.V157F	5	neg	pos (39%)	neg	pos (36%)	Biclon. M	n.d.	n.d.	neg
V33		0	neg	pos (81%)	pos (80%)	neg	UM	pos	pos	neg
V34		0	pos (50%)	neg	neg	pos (9,3%)	M	neg	neg	neg
V35		0	neg	pos (48%)	neg	pos (6%)	M	neg	neg	pos
V36	c.916C>T p.R306*	8	neg	n.d.	neg	pos (19%)	UM	pos	neg	neg
V37		0	neg	pos (70%)	neg	neg	Biclon. M	neg	pos	pos
V38		0	neg	pos (93%)	neg	neg	UM	pos	pos	pos
V39	c.758_764del6 p.I254_I255del	7	neg	neg	neg	pos (83%)	M	neg	pos	neg
V40	c.272_293del23 p.W91fs*49	4	neg	neg	neg	pos (10%)	M	pos	neg	neg
	c.630_631delCA p.T211fs*4	6								
V41	c.590T>A p.V197E	6	neg	neg	neg	neg	UM	pos	pos	neg
V42	c.590T>A p.V197E	6	neg	pos (30%)	neg	neg	M	neg	neg	pos

pos: positive; neg: negative; biclon: biclonal; M: mutated; UM: unmutated; n.d.: not determined

Cut-off for CD38 and CD49d: 30% of expressing cells; cut-off for ZAP70: 20% of expressing cells; cut-off for FISH (del13q, tris12, del11q, del17p probes): 5% of deleted nuclei

Percentage of FISH positive nuclei are reported within parentheses

Table S4. Validation cohort: evaluation "in blind" of 5 independent data anlyzers.

ID	*TP53* genotype	A1	A2	A3	A4	A5
**V1**	**del/mut**	D	D	D	D	D
**V2**	**mut/wt**	D	D	D	D	D
**V4**	**wt/wt**	N	N	N	N	N
**V5**	**mut/wt**	D	D	D	D	D
**V6**	**wt/wt**	D	D	D	D	D
**V7**	**wt/wt**	N	N	N	N	N
**V8**	**mut/wt**	D	D	D	D	D
**V9**	**del/mut**	D	D	D	D	D
**V10**	**del/mut**	D	D	D	D	D
**V11**	**wt/wt**	N	N	N	N	N
**V12**	**mut/wt**	D	D	D	D	D
**V13**	**mut/wt**	D	D	D	D	D
**V14**	**wt/wt**	N	N	N	N	N
**V15**	**wt/wt**	N	N	N	N	N
**V16**	**wt/wt**	D	D	D	D	D
**V17**	**wt/wt**	N	N	N	N	N
**V18**	**wt/wt**	N	N	N	N	N
**V19**	**wt/wt**	N	N	N	N	N
**V20**	**del/mut**	D	D	D	D	D
**V21**	**del/mut**	D	D	D	D	D
**V22**	**del/mut**	D	D	D	D	D
**V23**	**del/mut**	D	D	D	D	D
**V24**	**del/mut**	D	D	D	D	D
**V25**	**del/wt**	N	N	N	N	N
**V26**	**mut/wt**	D	D	D	D	D
**V27**	**mut/wt**	D	D	D	D	D
**V28**	**mut/wt**	D	D	D	D	D
**V29**	**mut/wt**	D	N	N	D	N
**V30**	**mut/wt**	D	D	D	D	D
**V32**	**del/mut**	D	D	D	D	D
**V33**	**wt/wt**	N	N	N	N	N
**V34**	**del/wt**	N	N	N	N	N
**V35**	**del/wt**	N	N	N	N	N
**V36**	**del/mut**	D	D	D	D	D
**V37**	**wt/wt**	N	N	N	N	N
**V38**	**wt/wt**	N	N	N	N	N
**V39**	**del/mut**	D	D	D	D	D
**V40**	**del/mut**	N	N	N	N	N
**V41**	**mut/wt**	D	D	D	D	D
**V42**	**mut/wt**	N	D	N	N	N

Colums A1-A5 indicate evaluations of the 5 independent data analyzers; D, dysfunction; N, normal.

Table S5: Characterization of CLL cases used for the comparison experiments.

ID	Match	Mutation	Exon	Tris12	Del13q	Del11q	Del17p	IGHV status	CD38	CD49d	ZAP70
C1	V16			pos (61%)	neg	neg	neg	M	neg	neg	neg
C2	V4			neg	neg	neg	neg	UM	neg	neg	neg
C3	T45			neg	neg	neg	neg	M	pos (63,8%)	neg	neg
C4	V15			neg	pos (85%)	pos (81%)	pos (5%)	UM	neg	neg	neg
C5	V19			pos (85%)	neg	pos (10%)	neg	UM	pos (98%)	pos (99,8%)	neg
C6	T64			neg	pos (12%)	pos (72%)	neg	Biclon. UM	pos (78,3%)	pos (41,1%)	pos (33%)
C7	T65			pos (15%)	pos (89%)	pos (80%)	neg	UM	neg	neg	pos (54%)
C8	V27	c.844C>T p.R282W	8	neg	pos (14%)	neg	neg	UM	pos (71,7%)	pos (98,4%)	pos (40%)
C9	V29	c.818G>A p.R273H	8	neg	n.d.	neg	neg	M	neg	neg	n.d.
C10	T19	c.724T>C p.C242R	7	neg	neg	neg	pos (14%)	UM	neg	pos (59,6%)	pos (20%)

pos: positive; neg: negative; biclon: biclonal; M: mutated; UM: unmutated; n.d.: not determined

Cut-off for CD38 and CD49d: 30% of expressing cells; cut-off for ZAP70: 20% of expressing cells; cut-off for FISH (del13q, tris12, del11q, del17p probes): 5% of deleted nuclei

Percentage of FISH positive nuclei are reported within parentheses

Table S6: Median fluorescence intensity values for TP53 and CDKN1A protein expression by FACS analysis

	TP53				CDKN1A			
	Untreated	Etoposide	Etoposide + Nutlin-3	Nutlin-3	Untreated	Etoposide	Etoposide + Nutlin-3	Nutlin-3
**EHEB**	85	135	383	236	342	932	1777	1048
**RAJI**	1427	2257	2176	2470	265	360	324	306
**MEC1**	218	293	323	214	284	290	311	288
**HL60**	94	122	126	108	151	140	144	166
**C1**	148	423	934	225	405	1548	2223	492
**C2**	124	489	1368	462	500	2890	3778	943
**C3**	12	474	805	92	40	1516	1838	225
**C4**	150	288	796	418	859	1087	4135	940
**C5**	220	612	2587	898	1075	2135	1440	1069
**C6**	5	20	513	164	4	38	132	11
**C7**	15	142	456	192	20	93	149	12
**C8**	5	104	115	50	2	18	12	7
**C9**	3	119	99	72	0	42	45	4
**C10**	17	299	439	137	58	151	126	68

Values are normalized by subtraction of isotype control

Table S7: Overview of the peculiar features of the different TP53 functional assays described by literature.

	Evaluation of TP53 and CDKN1A protein expression by western blot	Evaluation of TP53 and CDKN1A protein expression by FACS	Evaluation of *CDKN1A* expression levels by qRT-PCR	Evaluation of miR34a expression levels by qRT-PCR	Evaluation of TP53 target expression levels by RT-MLPA	Evaluation of TP53 protein expression by western blot and *CDKN1A* expression by qRT-PCR
**Method**	TP53 functionality is defined by evaluation of TP53 and CDKN1A protein expression by western blot at basal level and upon activation of DNA-damage pathway by irradiation or drug treatments	TP53 functionality is defined by evaluation of TP53 and CDKN1A protein expression by FACS at basal level and upon activation of DNA-damage pathway by irradiation or drug treatments	TP53 dysfunction is defined by low expression levels of *CDKN1A*	TP53 dysfunction is defined by low constitutive levels of miR34a	TP53 functionality is defined by evaluation of expression levels of the TP53 targets *CDKN1A*, *BAX*, *PUMA* and *CD95* by RT-MPLA at basal level and upon irradiation	TP53 functionality is defined by evaluation of TP53 protein expression by western blot at basal level and upon activation of TP53 by Nutlin-3. TP53 dysfunction is also defined by low expression levels of *CDKN1A* transcript.
**Advantages**	Using a combination of etoposide and Nutlin-3 to induce activation of DNA-damage pathway, defects of TP53 and ATM can be detected one from the other<br><br>Higher sensitivity of western blot detection	Using a combination of etoposide and Nutlin-3 to induce activation of DNA-damage pathway, defects of TP53 and ATM can be detected one from the other<br><br>Samples does not need to be purified also in the case of low percentages of CD19-positive cells	Using a combination of etoposide and Nutlin-3 to induce activation of DNA-damage pathway, defects of TP53 and ATM can be detected one from the other	Activation of TP53 pathway by DNA-damage using irradiation or drugs is not required	Using a combination of etoposide and Nutlin-3 to induce activation of DNA-damage pathway, defects of TP53 and ATM can be detected one from the other	Usage of non-genotoxic Nutlin-3 is safer for the operator<br><br>*CDKN1A* transcript evaluation by qRT-PCR is a more reliable readout of TP53 functional activity than CDKN1A protein levels by western blot<br><br>Higher sensitivity of western blot detection
**Disadvantages**	The assay does not consider the percentage of CD19-positive cells and samples need to be purified if this percentage is low (e.g. <70-80%)<br><br>Low TP53 disrupted clone sizes could not be revealed	Some problems could occur in the standardization of the method<br><br>Low TP53 disrupted clone sizes could not be revealed	The assay does not consider the percentage of CD19-positive cells and samples need to be purified if this percentage is low (e.g. <70-80%)<br><br>Low TP53 disrupted clone sizes could not be revealed	The assay does not consider the percentage of CD19-positive cells and samples need to be purified if this percentage is low (e.g. <70-80%)<br><br>Low TP53 disrupted clone sizes could not be revealed	The assay does not consider the percentage of CD19-positive cells and samples need to be purified if this percentage is low (e.g. <70-80%)<br><br>Low TP53 disrupted clone sizes could not be revealed	The assay does not consider the percentage of CD19-positive cells and samples need to be purified if this percentage is low (e.g. <70-80%)<br><br>Low TP53 disrupted clone sizes could not be revealed

Table S8. Characterization of CLL cases used for microarray experiments.

	Patient ID	GEO code*	*IGHV* status	FISH	ZAP-70 expression	CD38 expression	CD49d expression	*TP53* status
Gene expression profiling	GEP1	GSE18971	UM	13q-	pos	pos	pos	WT
	GEP2	GSE18971	UM	+12	pos	pos	neg	WT
	GEP3	GSE18971	M	+12, 13q-	neg	neg	pos	WT
	GEP4	GSE18971	UM	13q-	neg	pos	neg	WT
	GEP5	GSE18971	M	13q-	pos	neg	neg	WT
	GEP6	GSE18971	UM	+12	pos	neg	pos	WT
	GEP7	GSE18971	M	13q-	neg	neg	nd	WT
	GEP8	GSE18971	UM	+12	pos	pos	pos	WT
	GEP9	GSE18971	UM	13q-, 11q-	pos	neg	nd	WT
	GEP10	GSE18971	UM	+12	pos	pos	neg	WT
	GEP11	GSE18971	UM	normal	pos	pos	pos	WT
	GEP12	GSE18971	nd	nd	neg	neg	nd	WT
	GEP13	GSE18971	M	13q-	neg	neg	neg	WT
	GEP17	GSE18971	nd	17p-, 13q-	neg	nd	nd	c.476C>T; p.A159V; exon 5
	GEP18	GSE18971	UM	17p-, 13q-	pos	neg	nd	c.673-2A>G; intron 6
	GEP19	GSE18971	M	17p-, 13q-	neg	neg	nd	c.817C>A; p.R273S; exon 8
	GEP20	GSE18971	UM	+12, 17p-	pos	neg	nd	c.724T>C; p.C242R; exon 7
	GEP21	GSE18971	UM	17p-	pos	pos	60	c.517G>A; p.V173M; exon 5
	GEP22	GSE18971	M	17p-	neg	neg	nd	c.736_777del; p.M246_D259del; exon 7
	GEP23	GSE18971	UM	17p-, 13q-	pos	neg	nd	c.701A>G; p.Y234C; exon 7

m, male; f, female; M, mutated *IGHV*; UM, unmutated *IGHV*; normal FISH included cases with normal karyotypes, i.e. lacking 11q-, 13q-, 17p- and chromosome +12;

pos, positive, neg, negative, for ZAP70, CD38 and CD49d expression, according to Gattei et al (2008); nd not determined;

* identification number of CLL cases analysed by GEP, as reported at http://www.ncbi.nlm.nih.gov/geo/
