# Supplementary material for: Detection of TP53 dysfunction in chronic lymphocytic leukemia by an in vitro functional assay based on TP53 activation by the non-genotoxic drug Nutlin-3: a proposal for clinical application
Source: J Hematol Oncol. 2013 Nov 5;6:83. doi: 10.1186/1756-8722-6-83 (PMC4222122; doi:10.1186/1756-8722-6-83)
Supplement: Additional file 2 — Additional figures: Figure S1. Evaluation of apoptosis levels by Annexin V-7AAD and sensitivity of western blot analysis upon Nutlin-3 treatment. Figure S2. Western blot for CLL cases of the training cohort. Figure S3. Lack of the gene expression signature associated with Nutlin-3 treatment in TP53del/mut CLL samples. Figure S4. Western blot/qRT PCR assay for CLL cases of the validation cohort. Figure S5. Comparison with alternative TP53 functional assays using combinatorial strategies with etoposide. Figure S6. Comparison with evaluation of TP53 and CDKN1A protein expression levels by FACS analysis. [file 1756-8722-6-83-S2.pdf]

Supplemental Figure Legends

Figure S1. Annexin V-7AAD apoptosis assay by flow cytometry. A) Untreated cells were collected before treatment with Nutlin-3. “24h” and “48h” were collected, respectively, at 24 and 48 hours after treatment. Upper left quadrant, necrotic cells (Q1, annexin V^{low} and 7-AAD^{high}); upper right quadrant, dead cells (Q2, annexin V^{high} and 7-AAD^{high}); lower left quadrant, viable cells (Q3, annexin V^{low} and 7-AAD^{low}); lower right quadrant, early apoptotic cells (Q4, annexin V^{high} and 7-AAD^{low}). **B) Western blot of samples representing a dilution series of RAJI cells, expressing TP53 in basal conditions, mixed with EHEB cells, not expressing TP53 in basal conditions, with the proportion of 0%, 5%, 10%, 20%, 50% and 100% of RAJI cells.**

Figure S2. Western blot for CLL cases of the training cohort. Figure shows results from western blot assay of the 100 CLL cases of the training cohort subdivided in cases with a normal pattern (63 cases), cases with an intermediate pattern (19 cases) and cases with a mutant pattern (19 cases). The *TP53* genotype is indicated: *TP53* wild type (wt/wt), *TP53* deleted only (del/wt), *TP53* mutated only (mut/wt), *TP53* deleted/mutated (del/mut). The TP53 antibody detect a major band of 53 kDa and, in some cases (e.g. T23, T27, T34, T36, T38, T90), a minor band of 48 kDa. This second band may correspond to the alternatively spliced form, TP53β, described by Bourdon *et al* (2005). For cases showing a TP53 truncated form (e.g. T33, T69), the relative molecular weight is reported aside.

Figure S3. Lack of the gene expression signature associated with Nutlin-3 treatment in $TP53^{del/t}$ CLL samples. A) Heat-map generated with 144 genes found to be differentially expressed by supervised analysis between Nutlin-3 treated (red bar under the tree) samples versus the respective untreated (blue bar under the tree) counterparts, of $TP53^{wt/wt}$ CLL cases (13 cases), according to Zauli *et al* (2009) [1]. This signature was not shared by $TP53^{del/mut}$ CLL cases (7 cases), as indicated by the heat-map that was not able to split the treated (yellow bar under the tree) from the untreated (cyan blue bar under the tree) counterparts of $TP53^{del/mut}$ CLL cases. The colour scale identifies relative gene expression changes normalized by the standard deviation of 1 with 0 representing the mean expression level of a given gene.

Figure S4. Western blot/qRT PCR assay for CLL cases of the validation cohort A)Figure shows results from western blot assay of the 40 CLL cases of the validation cohort. The *TP53* genotype is indicated: *TP53* wild type (wt/wt), *TP53* deleted only (del/wt), *TP53* mutated only (mut/wt), *TP53* deleted/mutated (del/mut). The TP53 antibody detect a major band of 53 kDa and, in some cases (e.g. V6, V11, V20, V26, V29), a minor band of 48 kDa. This second band may correspond to the alternatively spliced form, TP53β, described by Bourdon *et al* (2005) [2]. For cases showing a TP53 truncated form (e.g. V12, V13, V20, V28, V36), the relative molecular weight is reported aside. B) Histograms show data, obtained by qRT-PCR amplification, of *CDKN1A* fold increase expression for each CLL case of the validation cohort.

Figure S5. Comparison with alternative TP53 functional assays using combinatorial strategies with etoposide. A) Figure shows results from western blot/qRT PCR assay for the evaluation of TP53 and *CDKN1A* expression on the 4 cell lines EHEB, MEC-1, RAJI and HL60 at basal level or upon the alternative treatments with etoposide, etoposide plus Nutlin-3 or Nutlin-3. B) Figure shows results from western blot/qRT PCR assay for the evaluation of TP53 and *CDKN1A* expression on the comparison series of 10 CLL cases composed by 4 11q deleted (11q-)/$TP53^{wt/wt}$, 1 $TP53^{del/mut}$, 2 $TP53^{mut/wt}$ and 3 $TP53^{wt/wt}$ cases at basal level or upon the alternative treatments with etoposide, etoposide plus Nutlin-3 or Nutlin-3.

Figure S6. Comparison with evaluation of TP53 and CDKN1A protein expression levels by FACS analysis. A) Histograms show results from FACS assay for the evaluation of TP53 and CDKN1A protein expression on the 4 cell lines EHEB, MEC-1, RAJI and HL60 at basal level or upon Nutlin-3 treatment. B) Histograms show results from FACS assay for the evaluation of TP53 and CDKN1A protein expression on the comparison series of 10 CLL cases composed by 4 11q deleted (11q-)/*TP53*$^{wt/wt}$, 1 *TP53*$^{del/mut}$, 2 *TP53*$^{mut/wt}$ and 3 *TP53*$^{wt/wt}$ cases.

Reference

1 Zauli G, di Iasio MG, Secchiero P, Dal Bo M, Marconi D, Bomben R, Del Poeta G, Gattei V: **Exposure of B cell chronic lymphocytic leukemia (B-CLL) cells to nutlin-3 induces a characteristic gene expression profile, which correlates with nutlin-3-mediated cytotoxicity**. *Curr.Cancer Drug Targets.* 2009, **9:**510-518.

2 Bourdon JC, Fernandes K, Murray-Zmijewski F, Liu G, Diot A, Xirodimas DP, Saville MK, Lane DP: **p53 isoforms can regulate p53 transcriptional activity**. *Genes Dev.* 2005, **19:**2122-2137.

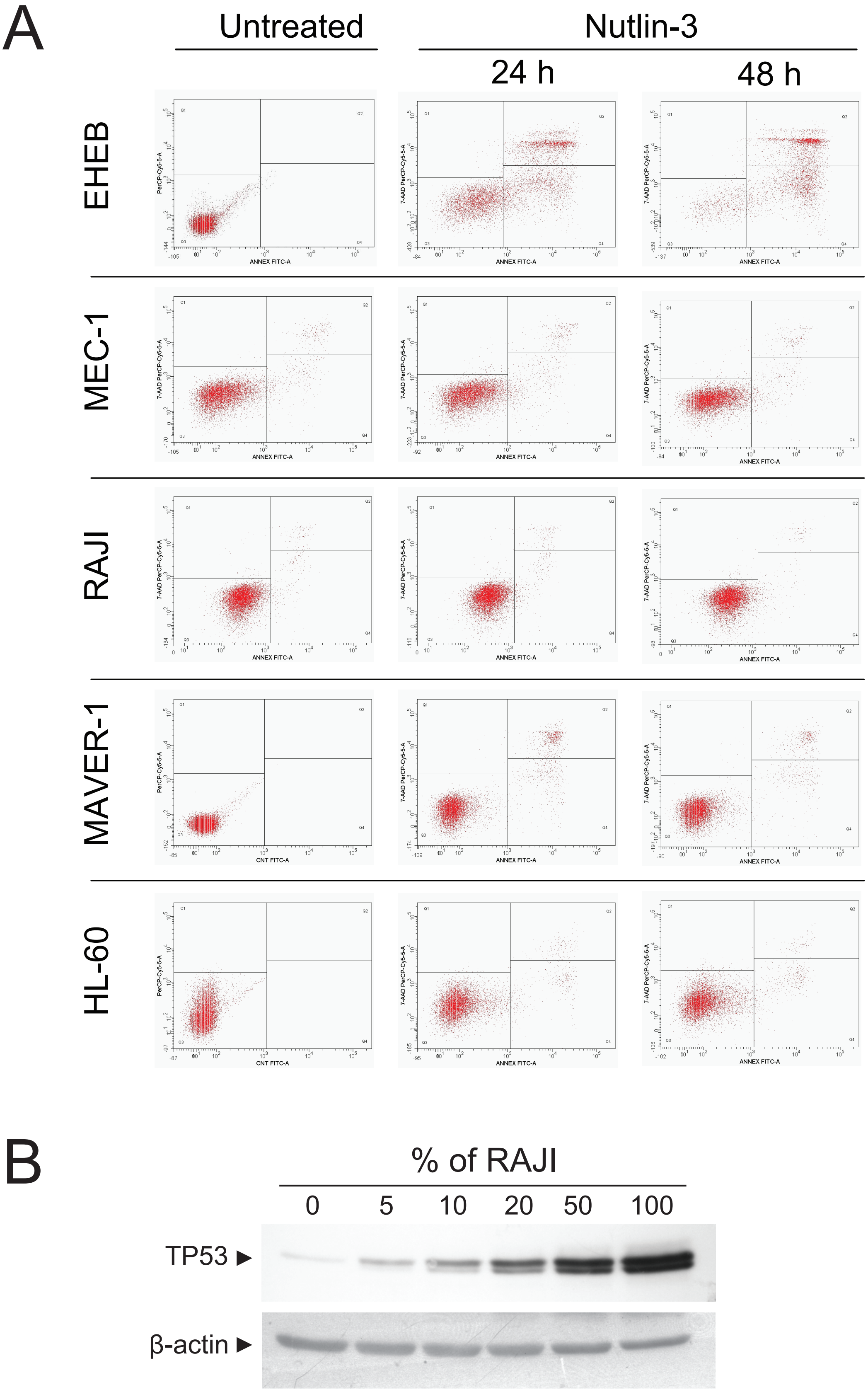
A
Untreated
Nutlin-3
24 h
48 h
EHEB
MEC-1
RAJI
MAVER-1
HL-60
B
% of RAJI
0
5
10
20
50
100
TP53
β-actin

Figure S1

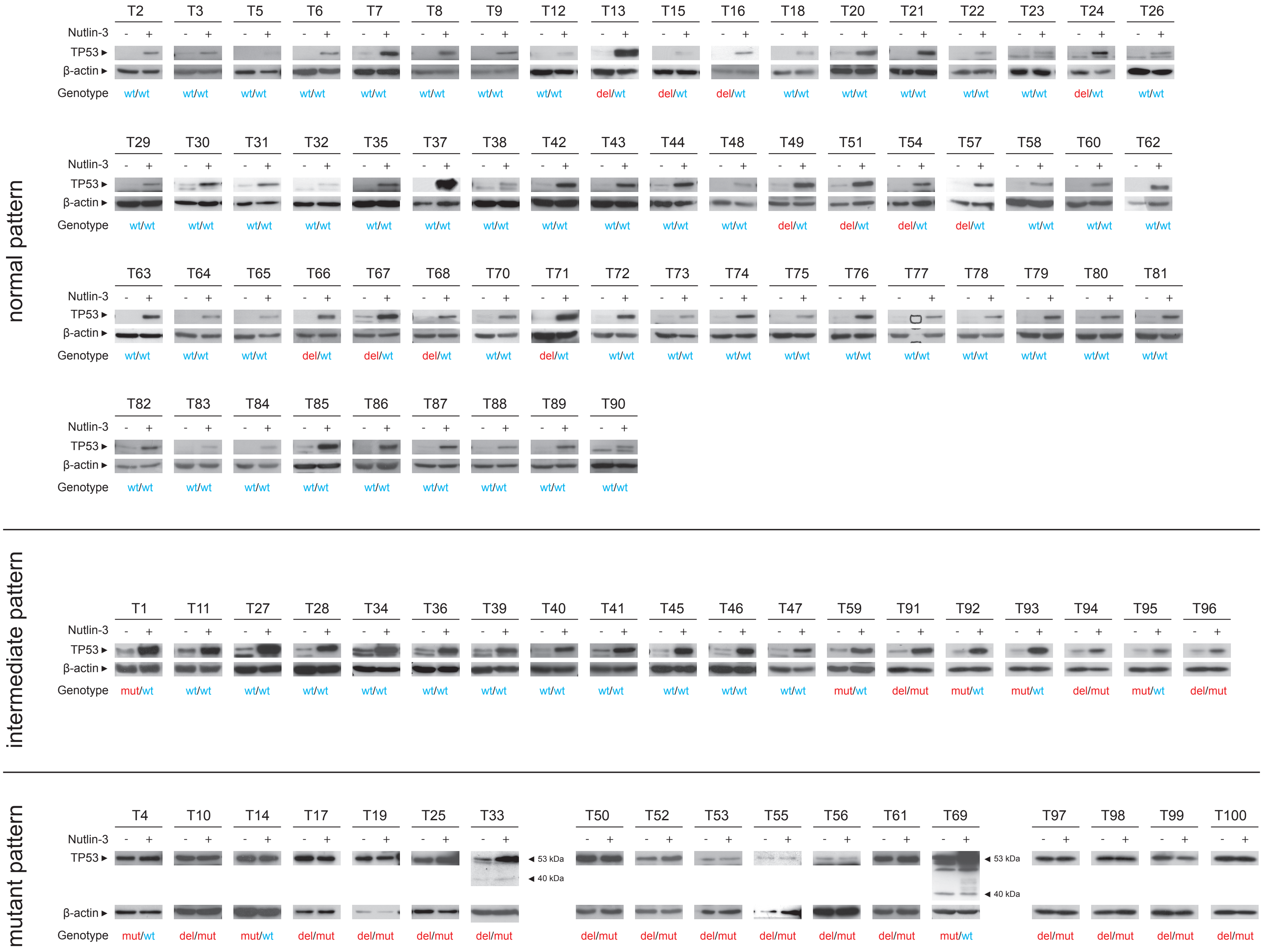
normal pattern
T2 T3 T5 T6 T7 T8 T9 T12 T13 T15 T16 T18 T20 T21 T22 T23 T24 T26
Nutlin-3
TP53
β-actin
Genotype
wt/wt wt/wt wt/wt wt/wt wt/wt wt/wt wt/wt wt/wt del/wt del/wt del/wt wt/wt wt/wt wt/wt wt/wt wt/wt del/wt wt/wt
T29 T30 T31 T32 T35 T37 T38 T42 T43 T44 T48 T49 T51 T54 T57 T58 T60 T62
wt/wt wt/wt wt/wt wt/wt wt/wt wt/wt wt/wt wt/wt wt/wt wt/wt wt/wt del/wt del/wt del/wt del/wt wt/wt wt/wt wt/wt
T63 T64 T65 T66 T67 T68 T70 T71 T72 T73 T74 T75 T76 T77 T78 T79 T80 T81
wt/wt wt/wt wt/wt del/wt del/wt del/wt wt/wt del/wt wt/wt wt/wt wt/wt wt/wt wt/wt wt/wt wt/wt wt/wt wt/wt wt/wt
T82 T83 T84 T85 T86 T87 T88 T89 T90
wt/wt wt/wt wt/wt wt/wt wt/wt wt/wt wt/wt wt/wt wt/wt
intermediate pattern
T1 T11 T27 T28 T34 T36 T39 T40 T41 T45 T46 T47 T59 T91 T92 T93 T94 T95 T96
mut/wt wt/wt wt/wt wt/wt wt/wt wt/wt wt/wt wt/wt wt/wt wt/wt wt/wt wt/wt mut/wt del/mut mut/wt mut/wt del/mut mut/wt del/mut
mutant pattern
T4 T10 T14 T17 T19 T25 T33 T50 T52 T53 T55 T56 T61 T69 T97 T98 T99 T100
53 kDa
40 kDa
mut/wt del/mut mut/wt del/mut del/mut del/mut del/mut del/mut del/mut del/mut del/mut del/mut del/mut mut/wt del/mut del/mut del/mut del/mut

Figure S2

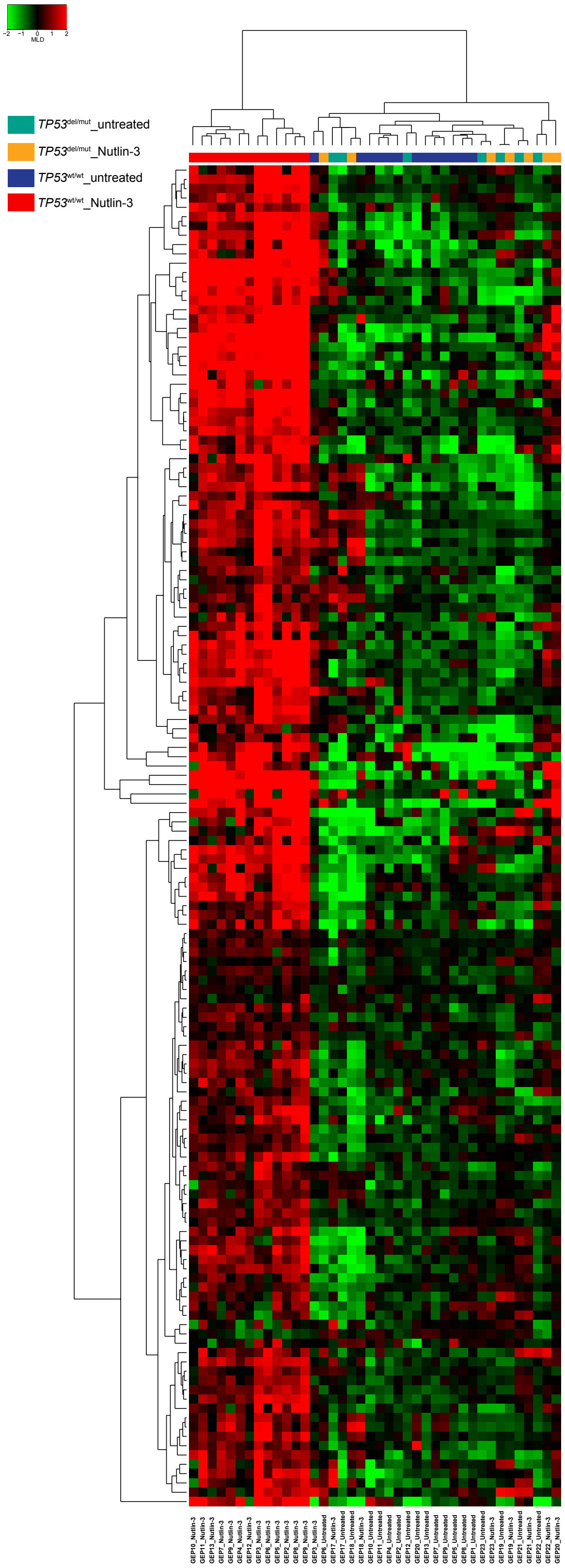

−2 −1 0 1 2
MLD
TP53del/mut_untreated
TP53del/mut_Nutlin-3
TP53wt/wt_untreated
TP53wt/wt_Nutlin-3
GEP10_Nutlin-3
GEP11_Nutlin-3
GEP13_Nutlin-3
GEP7_Nutlin-3
GEP9_Nutlin-3
GEP4_Nutlin-3
GEP12_Nutlin-3
GEP3_Nutlin-3
GEP6_Nutlin-3
GEP5_Nutlin-3
GEP2_Nutlin-3
GEP8_Nutlin-3
GEP9_Nutlin-3
GEP3_Nutlin-3
GEP6_Untreated
GEP17_Nutlin-3
GEP17_Untreated
GEP18_Untreated
GEP18_Nutlin-3
GEP10_Untreated
GEP11_Untreated
GEP4_Untreated
GEP2_Untreated
GEP12_Untreated
GEP20_Untreated
GEP13_Untreated
GEP7_Untreated
GEP9_Untreated
GEP5_Untreated
GEP8_Untreated
GEP1_Untreated
GEP23_Untreated
GEP23_Nutlin-3
GEP19_Untreated
GEP19_Nutlin-3
GEP21_Untreated
GEP21_Nutlin-3
GEP22_Untreated
GEP22_Nutlin-3
GEP20_Nutlin-3

Figure S3

A

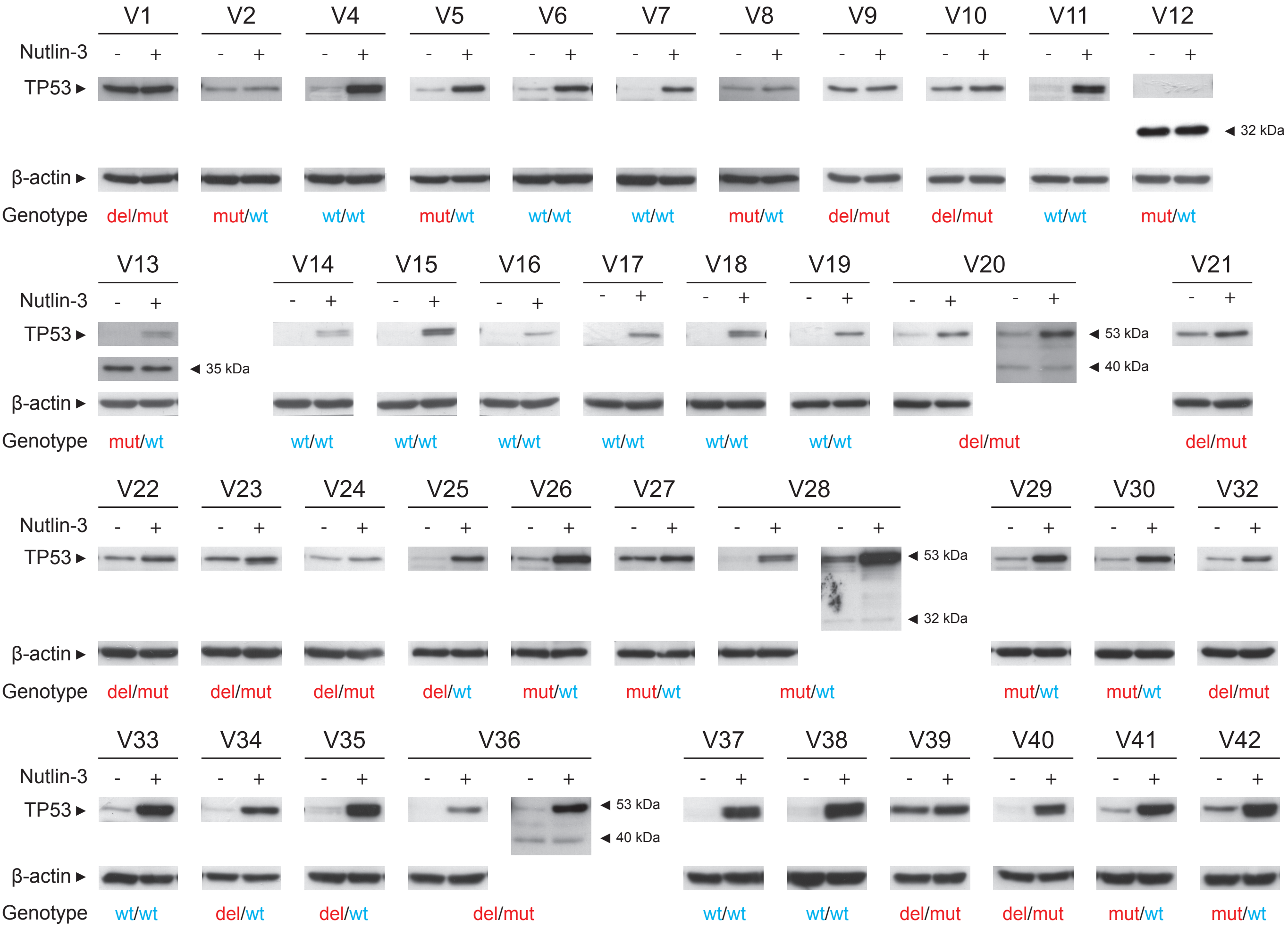
V1 V2 V4 V5 V6 V7 V8 V9 V10 V11 V12
Nutlin-3 - + - + - + - + - + - + - + - + - + - + - +
TP53
32 kDa
β-actin
Genotype del/mut mut/wt wt/wt mut/wt wt/wt wt/wt mut/wt del/mut del/mut wt/wt mut/wt
V13 V14 V15 V16 V17 V18 V19 V20 V21
Nutlin-3 - + - + - + - + - + - + - + - + - + - +
TP53
35 kDa
53 kDa
40 kDa
β-actin
Genotype mut/wt wt/wt wt/wt wt/wt wt/wt wt/wt wt/wt del/mut del/mut
V22 V23 V24 V25 V26 V27 V28 V29 V30 V32
Nutlin-3 - + - + - + - + - + - + - + - + - + - + - +
TP53
53 kDa
32 kDa
β-actin
Genotype del/mut del/mut del/mut del/wt mut/wt mut/wt mut/wt mut/wt mut/wt del/mut
V33 V34 V35 V36 V37 V38 V39 V40 V41 V42
Nutlin-3 - + - + - + - + - + - + - + - + - + - + - +
TP53
53 kDa
40 kDa
β-actin
Genotype wt/wt del/wt del/wt del/mut wt/wt wt/wt del/mut del/mut mut/wt mut/wt

B

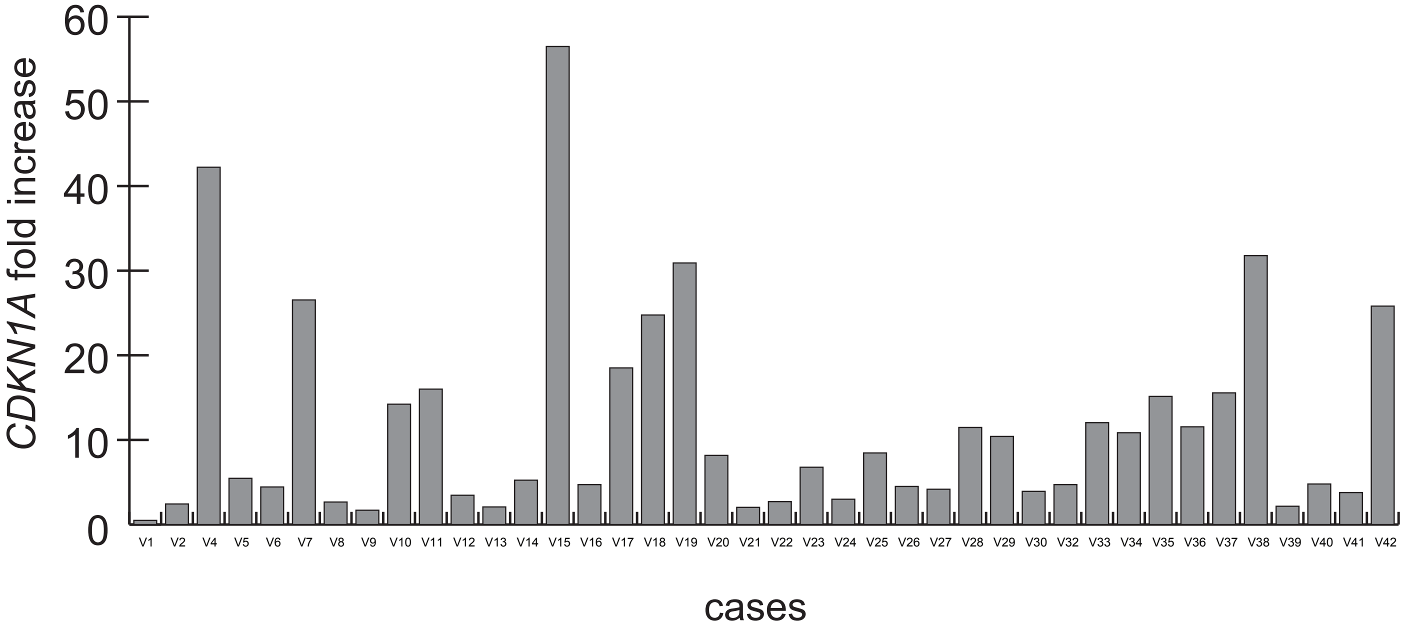
CDKN1A fold increase
0
10
20
30
40
50
60
V1 V2 V4 V5 V6 V7 V8 V9 V10 V11 V12 V13 V14 V15 V16 V17 V18 V19 V20 V21 V22 V23 V24 V25 V26 V27 V28 V29 V30 V32 V33 V34 V35 V36 V37 V38 V39 V40 V41 V42
cases

Figure S4

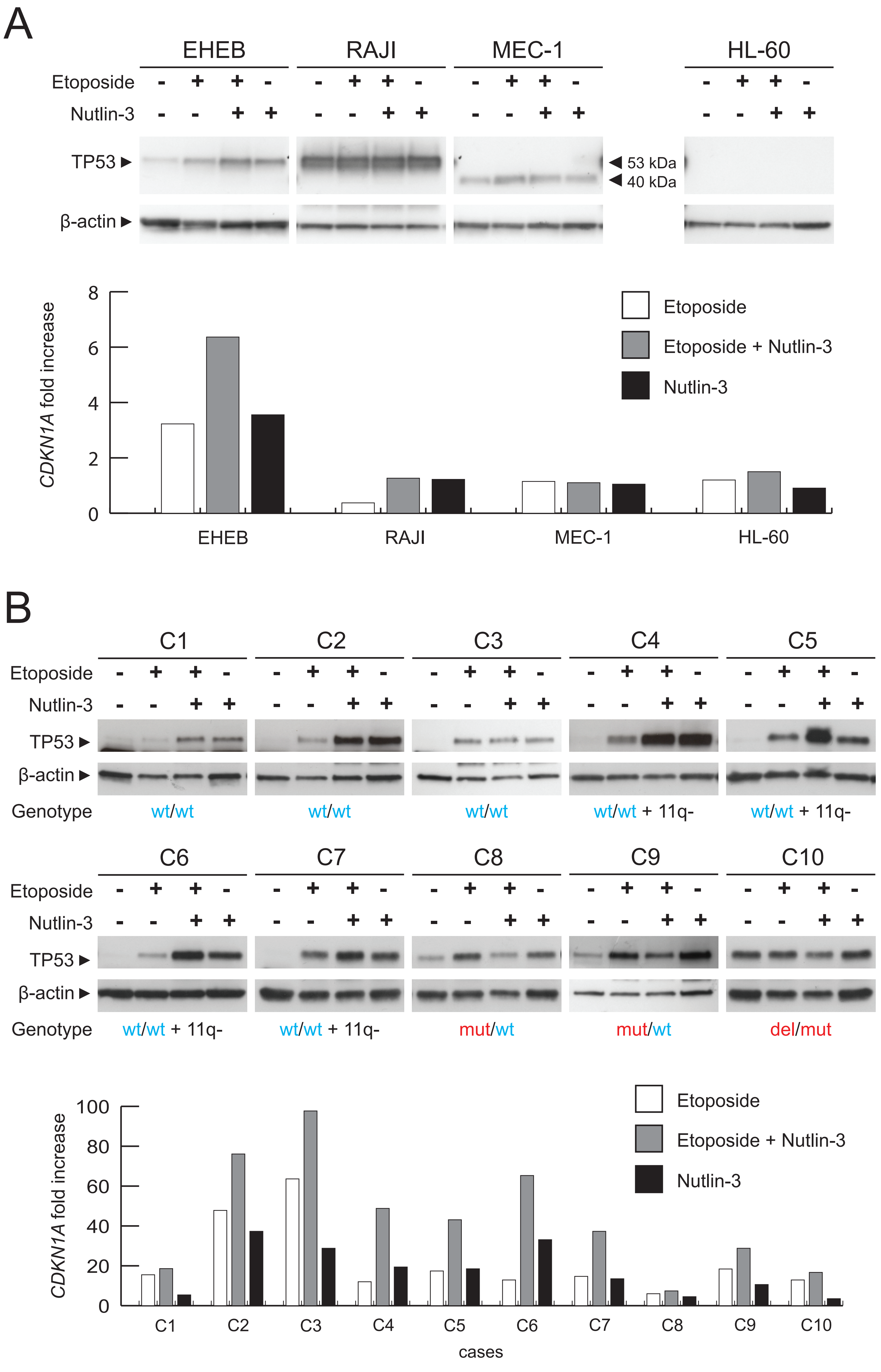
A
EHEB
RAJI
MEC-1
HL-60
Etoposide
Nutlin-3
TP53
β-actin
53 kDa
40 kDa
CDKN1A fold increase
Etoposide
Etoposide + Nutlin-3
Nutlin-3
B
C1
C2
C3
C4
C5
C6
C7
C8
C9
C10
Genotype
wt/wt
wt/wt + 11q-
mut/wt
del/mut
cases

Figure S5

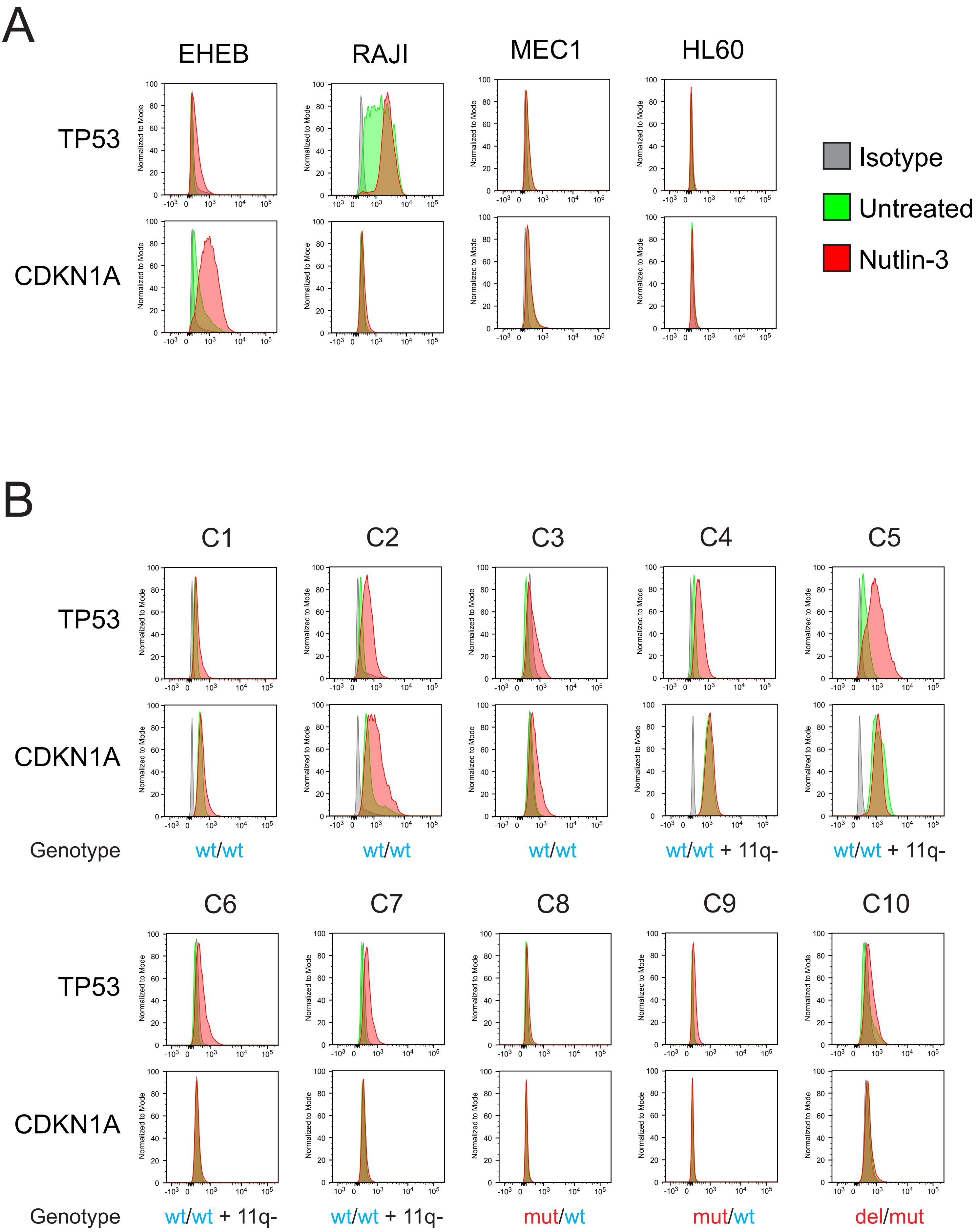
A
EHEB
RAJI
MEC1
HL60
TP53
CDKN1A
Isotype
Untreated
Nutlin-3
Normalized to Mode
B
C1
C2
C3
C4
C5
C6
C7
C8
C9
C10
Genotype
wt/wt
wt/wt + 11q-
mut/wt
del/mut

Figure S6
